# Testing the Capacity of a Multi-Nutrient Profiling System to Guide Food and Beverage Reformulation: Results from Five National Food Composition Databases

**DOI:** 10.3390/nu9040406

**Published:** 2017-04-21

**Authors:** Emilie Combet, Antonis Vlassopoulos, Famke Mölenberg, Mathilde Gressier, Lisa Privet, Craig Wratten, Sahar Sharif, Florent Vieux, Undine Lehmann, Gabriel Masset

**Affiliations:** 1 Human Nutrition, School of Medicine, College of Medical, Veterinary and Life Sciences, University of Glasgow, Glasgow G31 2ER, UK; emilie.combetaspray@glasgow.ac.uk (E.C.); Craig.wratten@live.co.uk (C.W.); saharsharif3@gmail.com (S.S.); 2 Nutrient Profiling, Public Health Nutrition, Nestlé Research Center, Vers-chez-les-Blanc, 1000 Lausanne 26, Switzerland; famke.molenberg@gmail.com (F.M.); mathilde.gressier@rdls.nestle.com (M.G.); undine.lehmann@rdls.nestle.com (U.L.); gabriel.masset@rdls.nestle.com (G.M.); 3 Division of Human Nutrition, Wageningen University, 6700AA Wageningen, The Netherlands; 4 MS-Nutrition, 13005 Marseille, France; lisa.privet@ms-nutrition.com (L.P.); florent.vieux@ms-nutrition.com (F.V.)

**Keywords:** nutrient profiling, food supply, reformulation, food composition database

## Abstract

Nutrient profiling ranks foods based on their nutrient composition, with applications in multiple aspects of food policy. We tested the capacity of a category-specific model developed for product reformulation to improve the average nutrient content of foods, using five national food composition datasets (UK, US, China, Brazil, France). Products (*n* = 7183) were split into 35 categories based on the Nestlé Nutritional Profiling Systems (NNPS) and were then classified as NNPS ‘Pass’ if all nutrient targets were met (energy (E), total fat (TF), saturated fat (SFA), sodium (Na), added sugars (AS), protein, calcium). In a modelling scenario, all NNPS Fail products were ‘reformulated’ to meet NNPS standards. Overall, a third (36%) of all products achieved the NNPS standard/pass (inter-country and inter-category range: 32%–40%; 5%–72%, respectively), with most products requiring reformulation in two or more nutrients. The most common nutrients to require reformulation were SFA (22%–44%) and TF (23%–42%). Modelled compliance with NNPS standards could reduce the average content of SFA, Na and AS (10%, 8% and 6%, respectively) at the food supply level. Despite the good potential to stimulate reformulation across the five countries, the study highlights the need for better data quality and granularity of food composition databases.

## 1. Introduction

The capacity for nutrient profiling, to rank or classify foods based on their nutrient composition [1], has motivated recent research on the relationship between foods, their nutrient composition, and potential diet and health outcomes [2,3,4,5,6,7,8]. Many nutrient profiling systems have been designed, ranging in purpose including front-of-pack (FOP) labelling to guide food selection decisions [9,10,11,12] and the regulation of marketing to children [13,14]. A review of all available nutrient profiling systems is currently being carried out [15]. For some systems, reformulation is the primary focus, mainly food industry developed systems [16,17], while, for others, food reformulation is a secondary outcome—for example, systems designed for FOP labelling and food procurement regulation [18,19]. Thus far, the majority of initiatives with a primary focus on reformulation, either voluntary or mandatory schemes, adopt a single nutrient approach, most often focused on sodium [20,21,22], instead of nutrient profiling. The Nestlé Nutritional Profiling System (NNPS) [16] and the similar Unilever system [17] are applied within the respective food companies to guide their specific reformulation actions. These two systems provide targets for the simultaneous reduction of nutrients like sodium, sugars, saturated fat and trans fat, and, in the case of the NNPS, it also provides targets for the minimum content of protein, fibre and calcium. To date, however, little is known regarding the external relevance of such systems (beyond company portfolios) to improve the average nutritional composition of foods. 

This study aimed to test whether the category-specific and multi-nutrient Nestlé Nutritional Profiling System (NNPS), developed for product reformulation, was suitable to identify reformulation priority areas, across several countries with contrasting eating habits and levels of industrialisation (developed countries vs. rapidly developing countries in nutrition transition) [23]. 

## 2. Materials and Methods 

### 2.1. Food Composition Datasets and Nutrient Profiling System

National food composition datasets were obtained from the USA [24], the UK [25], Brazil [26], China [27] and France [28]. 

The Nestlé Nutritional Profiling System (NNPS) is a category-specific system that calculates nutrient targets per serving as consumed, based on age-adjusted dietary guidelines, as previously explained [16]. The NNPS outcome is dichotomous (pass/fail), requiring all nutrient targets to be met to obtain a positive/pass outcome. For this analysis, the adult target values were used for energy, total fat, saturated fat, added sugar and sodium (nutrients to limit, applied in all food categories); and fibre, calcium and protein (nutrients to encourage, applied in specific food categories). These nutritional factors were retrieved from the five food composition datasets. A full description of each category represented in the NNPS has been reported previously [16].

### 2.2. Categorisation and Inter-Rater Agreement

The NNPS is designed to only assess foods amenable to reformulation (FARs), i.e., all food and beverage products that have undergone some form of processing with substantial impact on their ingredients and/or nutrient composition. For example, frozen or canned vegetables without the addition of salt would not be considered as FARs, but a frozen salted vegetable stir-fry would be included in the analysis. Since all databases did not allow for differentiation between packaged foods, take-away foods and home-prepared meals, all mixed (composite) dishes and prepared meals were considered as FARs (noting the potential relevance of nutrient profiling to regulate dishes prepared in collectivities).

In each food composition dataset, foods and beverages were either excluded from the analyses (non-FARs, out of scope of the NNPS) or categorised into one of the 35 categories of the NNPS, by four trained researchers. When categorisation was not straightforward, allocation to a specific category was reviewed by the team. Items which did not belong to any category after this step were considered as out of scope (e.g., frozen fruit/vegetable, pure coffee, uncooked meat/fish).

To assess the degree of categorisation agreement between researchers, a random subsample of the food composition datasets was evaluated (random 10% of each database). One researcher at each centre (*n* = 3) re-categorised all products in the subsample. The percentage of food products allocated to the same category by all researchers was calculated; and the Cohen’s kappa coefficient was generated for each dataset.

### 2.3. Missing Data and Imputation

In all five datasets, data for energy (kcal), protein, fibre, calcium, sodium, total fat, saturated fat, and total sugar were provided per 100 g. 

There were no missing data in the US and French datasets, except for the estimated added sugars variable, specifically consolidated for the respective national dietary surveys (National Health and Nutrition Survey—NHANES, and Enquête Individuelle et Nationale des Consommations Alimentaires—INCA2, respectively), as described below. To account for missing nutrient values in the other datasets, all products with a missing nutrient value (relevant to any specific category) were excluded, except when this represented over 50% of the entire dataset, which was the case for added sugars (all datasets) and saturated fats (Chinese dataset). 

Any missing data carried forward were addressed in one of the following ways. Added sugars were either calculated using the Food Pattern Equivalent Database [29], for the US database, or were estimated by summing the amount of each assimilated sugar ingredients, based on the methodology proposed by Louie et al. and using a national average food recipes database [30], for the French dataset. In the case of the UK dataset, added sugars were derived using an algorithm building on the available total sugar, lactose, maltose and galactose values (the full algorithm is outlined in Supplementary Figure S1). In the Brazilian dataset, added sugars were provided directly as a variable, so no attempt was made to estimate or calculate any missing data. Finally, added sugars and missing data for saturated fats in the Chinese dataset were imputed via linkage to the US database [31]. 

In a final step, if missing data still existed in the datasets, they were set to the maximum NNPS target for the relevant nutrient.

### 2.4. NNPS Calculation

The NNPS algorithm was applied using the 3.2.3 R package (R Foundation for Statisticcal Computing, Vienna, Austria) for the UK, Brazilian, Chinese and US datasets; and SAS 9.4 (SAS Institute, Cary, NC, USA) was used for the French database. As there are no internationally agreed serving sizes and such regulations are lacking even in local/regional levels, the portion sizes used in this analysis were the recently updated US RACCs (reference amounts customarily consumed) to allow cross-country comparisons [32]. A product was given an NNPS overall pass only if every single relevant nutrient target was met, as the algorithm is non-compensatory. 

### 2.5. NNPS Outcome Analyses

Only FARs (referred to as foods and beverages, or products, hereafter) were included in the analysis, with all raw/unprocessed products excluded. 

Descriptive statistics were used to analyse the NNPS outcomes. The overall NNPS pass rate and nutrient-specific pass rates (number of products that pass, over total number of FARs) were calculated for each of the five countries and for each separate category. The pass rate per country was calculated by dividing the total number of products that met every single relevant nutrient criterion by the total number of categorised products in each country. The pass rate per category was calculated by dividing the number of products that met every single relevant nutrient criterion by the total number of products in each category.

Differences across countries of both global and nutrient specific pass rates were tested using the chi-square tests. When data from all countries were collated, weighted averages were calculated based on the number of products in each country. Weighted averages were always presented alongside the min–max range of pass rates/reductions among all five countries. 

A modelling scenario was designed to assess the potential impact of reformulation to NNPS targets on the average nutrient composition of products: the composition of all “NNPS Fail” products was changed to the threshold NNPS targets for each nutrient above the target. The potential change in nutrient composition of “NNPS Fail” products as well as all foods and beverages (whole supply) were assessed.

## 3. Results

### 3.1. Food Composition Tables and Data Completeness

Data quality, in terms of granularity and completeness for nutrient content, was variable among datasets. The US database (FNDDS) was the most detailed with 3135 categorised FARs (41% of the total US dataset), followed by the UK (*n* = 1527, McCance and Widdowson Composition of Foods). The Chinese dataset focused largely on raw/unprocessed ingredients, with only 34% of the products being classified as FARs. The French database, on the other hand, included a high proportion of FARs with 68% of the total French dataset (*n* = 913) categorised as such (Table 1). Data completeness in the categorised products was high for the US and French datasets. Added sugars, although provided in the Brazilian dataset, had very low completeness (33% of the categorised and analysed products). SFA content was the most limited nutrient in terms of completeness in the Chinese dataset, with 40% of all FARs containing such information. 

### 3.2. Representativeness of NNPS Food Categories for Each National Dataset 

A total of *n* = 7183 food and beverage products were categorised as FARs and analysed across all five countries. The Chinese dataset had the least number of populated categories (*n* = 27), whereas the US had the most (*n* = 34) (Table 1, and Supplementary Table S1). 

The distribution of food items per category varied, depending on the country. Across all five datasets, 24 “minor” categories consistently represented less than 5% of the total number of products for each individual country. The remaining “main” 11 categories represented 71% (US) to 83% (Brazil) of all the products in each national dataset: complete meals; meat/fish/replacers; small meals; side dishes; cold cuts and spreads; cheeses; yoghurts and fresh cheeses; milk-based beverages; cakes, cookies and desserts; beverages; and dressings. The meat/fish/replacers as the center of plate category were consistently the largest in all five countries, except France—from 16% of products in the US dataset to 40% of products in the Brazilian dataset. The largest category in the French dataset was cheeses (11%). The milk-based breakfast beverages category was the smallest category in the UK database (0.2%) and was not populated in the other four datasets. Five categories (milk-based breakfast/enriched/cereal-based beverages, water ices and sorbets and mayonnaise) contained less than 10 items in each and every dataset. The enriched beverages category was not populated in any of the datasets. 

Only three categories consistently represented more than 5% of products in each national dataset, across all datasets: meat/fish/replacers (9%, France–40%, Brazil), side dishes (7%, France–17%, UK) and cakes, cookies and desserts (9%, UK–13%, China). 

### 3.3. Inter-Rater Agreement Scores

Average inter-rater agreement between research centres, for product allocation to NNPS categories, ranged from 51% (US) to 88% (Brazil) (Table 2). The kappa statistic [33] for each of the five datasets by the three reviewers ranged from substantial agreement (0.62 in the US data) to almost perfect agreement (0.87 in the Brazilian data) (Table 2).

### 3.4. NNPS Outcome—Overall Results

**Figure 1:**
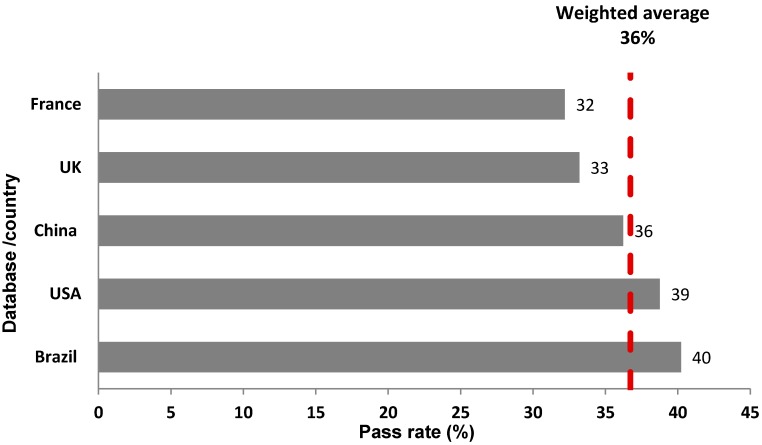
Overall pass rate (proportion of products meeting the Nestlé Nutritional Profiling System criteria) across all databases.

The overall mean pass rate across all five countries showed that approximately a third (36%) of all products achieved the NNPS standards, with significant differences between countries (*p* < 0.001) (Figure 1). The overall pass rate per country ranged from 32% (France) to 40% (Brazil). 

Products not passing the NNPS standards was often due to two or more nutrients not meeting their specific criteria (Table 3). Indeed, 55% to 69% of products (US and France, respectively; average 605) did not meet two nutrient criteria or more. A small proportion of products in every single dataset, averaging 14% (ranging from 12% to 15% in China and Brazil, respectively), did not meet the criteria for three nutrients or more.

Across all five datasets, the nutrients most often above respective NNPS criteria were saturated fat (23%–44%) and total fat (31%–47%). There were large variations in the proportion of products not meeting the calcium standard (from 22% in the UK to 57% in Brazil) and protein (from 4% in France to 25% in the UK) (Table 3).

### 3.5. NNPS Outcome by Category

**Figure 2:**
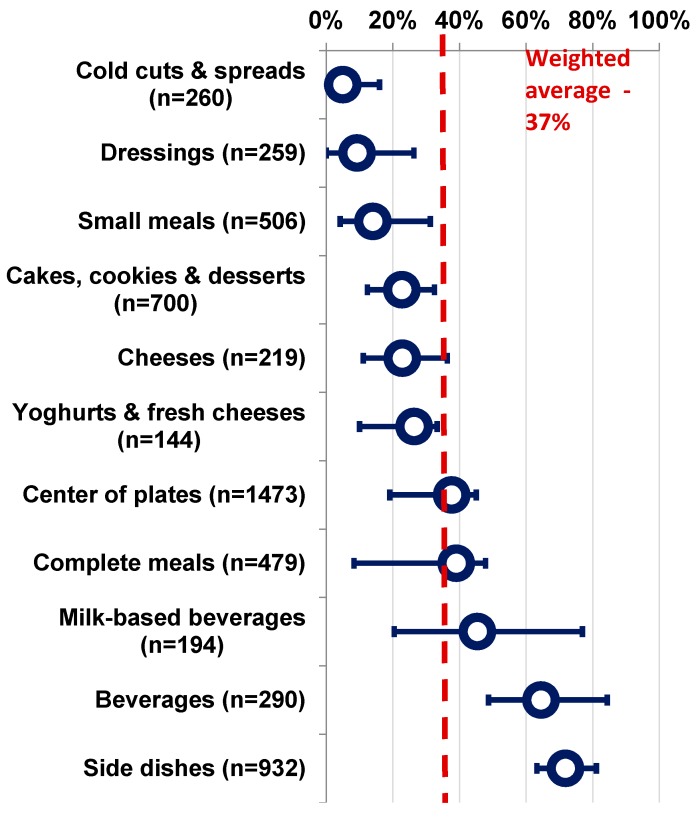
Pass rate for the 11 major categories (proportion of products meeting the NNPS criteria) across all databases (weighted average, min, max).

All categories, for all countries, contained products that did not meet at least one of the NNPS nutrient requirements (Supplementary Table S2a–e). The following analysis only focused on the 11 “main” categories, i.e., those representing at least 5% of any country’s database, to ensure that the findings were relevant to well populated categories. Weighted average pass rates for individual categories ranged from 5% to 72% (weighted average across the 11 main categories, 37%), with large inter- and intra-category variability (Figure 2). Low variability was documented in categories with low pass rates such as cold cuts and spreads (2%, France–16%, China), and higher pass rate such as the side dishes category (63%, UK–82%, Brazil). Higher variability in the pass rate was noted in the milk-based beverage category (20%, China–77%, UK) and the complete meals category (8%, China–48%, France).

Overall, most categories had pass rates lower than 50%. The detailed pass rates per category and per country are provided as supplementary Table S2a–e. The detailed average nutrient content comparisons between NNPS pass/fail products per category and per country are provided as supplementary Table S3a–e.

### 3.6. Achieving NNPS Standards—Impact of Modelled Reformulation on Average Nutrient Content

**Figure 3:**
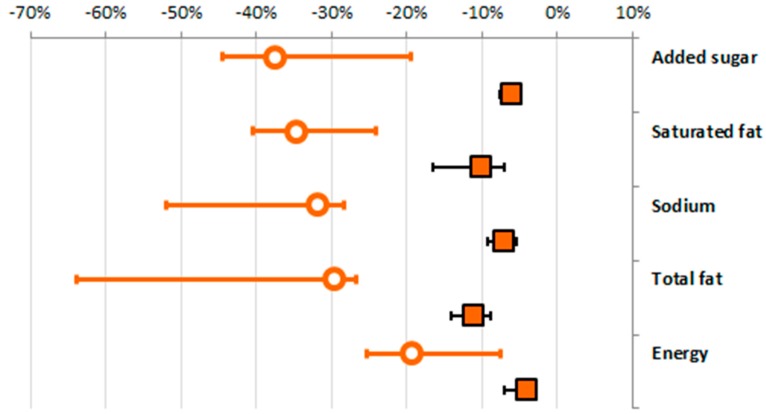
Average minimum change (%) required in NNPS fail products to achieve NNPS standards (circle), and overall modelled change (%) in all products amenable to reformulation (squares), across all five national datasets (weighted average, min, max). Circles: change required in NNPS Fail products for the specific nutrient; squares: overall change in the average nutrient content across all products analysed.

In the modelling scenario, the NNPS targets were used as proposed manufacturing standards, hence all “NNPS fail” products were speculatively “reformulated” to meet the NNPS targets. As shown in Figure 3, 1095 to 2508 products were identified as to be reformulated for nutrients to limit with weighted average reductions between 19% and 38%. The same holds true for the nutrients to encourage, where “NNPS fail” products would require average increases of fiber (177%), protein (95%), and calcium (607%) to meet NNPS targets, but the absolute number of products to be reformulated was only 36, 498 and 216, respectively (data not shown). 

These changes in the composition of the “NNPS fail” products would then translate to an average reduction of 11% for total fat, 10% for saturated fat, 8% for sodium, 6% for added sugar, and 4% for energy in all FARs (Figure 3). Small increases would be achieved for protein (7%) and calcium (18%), while little impact would be seen for fibre (<1%; criterion only relevant in two, low populated, categories). 

Table 4 and Table 5 show the product categories that would require the largest reformulation per nutrient in % reduction/increase, with high variability documented both between countries and categories. Details of the reduction/increase required per category per country in all “NNPS fail” products are provided in Supplementary Table S4a–e. It is also important to highlight that % reductions/increases did not always reflect the absolute changes, i.e., similar 30% reduction in total fat in dressings and culinary sauces would translate in 6g and 4g reductions in absolute values, respectively—a reflection of the fat carriers in the food supply and different serving sizes. The same holds true for all nutrients to limit and nutrients to encourage (Table 4 and Table 5). 

## 4. Discussion

Nutrient profiling systems are intended to be actionable tools for public health policies and initiatives, and their practical applicability is an important feature in their development and validation process. Beyond scientific validity, there is a need to evaluate whether nutrient profiling systems can be effectively implemented, as recently highlighted by the French Food Safety Agency [34]. The ease of implementation has been judged on three main aspects: categories, reference amounts and nutrients. 

Here, we tested whether the NNPS categories, reference amounts, nutrients and respective nutrient targets, originally linked to the Nestlé portfolio, were (i) applicable in the food composition databases of five countries, and (ii) suitable to identify product categories to prioritize for reformulation. 

### 4.1. Application to Food Composition Databases 

The number of NNPS categories populated with at least 10 products varied between countries (from 14 to 29 out of 35), suggesting that most NNPS categories were relevant to the global food supply. There was substantial classification agreement between the three research centers, as measured by the kappa statistic. However, categories such as “complete meals”, “side dishes”, “small meals”, and “Asian noodles” still presented classification challenges (data not shown) as the possibility of an overlap was high between categories, due to characteristics not inherent to the food composition datasets (e.g., noodles consumed as a complete meal, a side dish especially in China, a small meal in some countries; especially the dried noodles with sauces—pot noodles in Europe or ramen noodles in the USA or even a soup if more water is added). These factors may have influenced the inter-rater agreement score, as classification to these categories highlighted difficulty in the decision-making, and imply that a good understanding of both the NNPS and the local food supply are required prior to its application. This issue was more prominent in the US dataset (the largest in this analysis) suggesting that classification becomes more challenging as decision making demands increase (absolute number of products combined to number of potential categories for allocation). The inclusion of too many product categories has been criticized [35,36,37], as it could possibly make system implementation more difficult, especially across different countries. Interestingly, although the NNPS has a high number of categories (*n* = 35), sodium reduction initiatives also tend to have a long list of product categories, e.g., 95, 158, and 76 categories contained in the Health Canada, the US Food & Drugs Administration, and the UK responsibility deal reformulation initiatives, respectively [20,21,38]. This added complexity allows for better consideration of the constraints associated with reformulation (i.e., technical limitations in the reformulation process, baseline nutrient content of the foods to be reformulated, and intrinsic nutrient profiles per product category). 

A key observation of this analysis is that applying Nutrient Profiling systems to national food composition databases raises several challenges. The datasets used in this study showed variable data completeness, which called for data imputation strategies. Nutrient profiling systems and regulatory documents set sugar content targets either based on added sugar [14,39] (like the NNPS), free sugar [40] or total sugar content [9]. Total sugar was the most commonly found variable in the datasets we used, while added sugars could be calculated using specific analysis techniques (as carried out for the US and French datasets). Only the Brazilian dataset provided added sugar content, albeit with very limited data completeness: a large proportion of products lacked added sugar values (67%). The UK was a unique case as it provided total sugar content as well as a detailed breakdown of mono and disaccharides. Given this detailed breakdown of sugars, it was possible to develop an algorithm to determine added sugar content. Added sugars, as well as free sugars, can only be calculated and not analytically determined, hence only such a detailed representation of all sugar subclasses could sufficiently support policy makers in developing and validating added/free sugar reduction targets. This is not the case for most datasets currently available. Updating standard/generic food composition datasets to include higher quality data, having more regular updates of the nutrient content, and increasing data granularity should be a key focus for future research. Another option, in the case of sugars, would be to regulate the labelling of added sugar content, as recently proposed by the US FDA [41]. Initiatives are also currently being developed to standardize food composition databases, which is a promising way forward in addressing data quality issues [3]. To improve data granularity, further initiatives are needed to explore the potential of datasets containing nutritional labels information (e.g., the Oqali dataset in France, the Global New Product Database, and the US Department of Agriculture branded food composition dataset [42,43,44]) in dietary research and policy making. These datasets offer the potential to be linked with sales data to obtain a better understanding of purchase habits and tailor nutrition policies to products consumed [45,46]. In this analysis, branded food composition datasets were not used—such existing datasets [43] may include a large amount of products but their representativeness of the foods sold in respective countries remains difficult to judge. Moreover, most existing branded datasets are not available in open access. Meanwhile, crowd sourced datasets [47], usually open access, may not offer the details nor the data quality needed to conduct research and guide decisions. 

### 4.2. Global Applicability and Potential to Stimulate Reformulation

The global applicability of the system was tested by comparing the system’s performance in five countries with different eating habits, levels of industrialisation and local taste preferences, a factor that can substantially influence the content of taste-carrying nutrients like salt [34]. Across the five datasets, most products (63%) were identified as potential targets for reformulation. In the reformulation scenario, the NNPS showed greater capacity to identify categories that would need decreases in nutrients to limit—included in all food categories—than increases in nutrients to encourage—which are included in a few categories only. In selected categories and countries, the system did show specific potential to improve the calcium and protein contents of some dairy-based products, or help identify products that were not ‘truly’ dairy-based, i.e., with milk being the main ingredient. Thereby, the NNPS could be judged as setting relevant targets for reformulation that are achievable, but would nonetheless require most products to be reformulated to some extent. The modelled nutrient reductions are in the range of 6%–10%, which is known to fall within organoleptic acceptance [48,49] and hence the NNPS could support a stepwise reduction in nutrients like salt and sugar.

There was high intra- and inter-country heterogeneity in pass rates. This heterogeneity could be explained by multiple factors linked either to the system itself or to the food composition databases used. One such factor is data completeness and granularity in the datasets.

The datasets used showed variable data completeness and at times a skewed representation of the raw/unprocessed foods over composite dishes, or vice versa. As an example, between 34% and 68% of all products were identified as amenable to reformulation. As such, our results need to be evaluated with caution; and between-countries differences in nutrient composition may reflect both true differences linked to consumer preferences: for example, UK soups contained on average four times the salt of those in the Chinese dataset (0.4 vs. 0.1 g salt per 100 g, respectively); meanwhile, pizzas in Brazil contained twice the amount of salt of those in the UK dataset (0.7 vs. 0.3 g salt per 100 g, respectively) [34]. These findings are limited by the fact that food composition tables lack granularity. Although this is a caveat of this study, it would be an inherent caveat of any food policy decision based on similar, national data. Previous reports have identified larger numbers of products by direct sampling of the UK market, compared to the sample described herein [50,51,52], and such an issue would be better addressed through comprehensive branded food databases, as mentioned earlier. 

A key hypothesis behind the NNPS is that multi-nutrient non-compensatory (all nutrient targets must be met simultaneously) reformulation strategies are more suitable to improve the overall nutritional profile of the food supply as opposed to approaches focused on single nutrients. In this analysis, the non-compensatory algorithm of NNPS explained the large proportion of NNPS Fail products. In most categories, two or more nutrients were identified as not meeting the NNPS target in approximately half of the analyzed products. Categories with multiple limiting nutrients showed lower pass rates as opposed to those with one or two limiting nutrients. For example, in comparison to a single nutrient system like the US FDA [38] or the National Salt Reduction Initiative [53] sodium reduction targets, the NNPS pizza category would indeed set more lenient targets in single nutrients like sodium (566 mg/100 g vs. 490–500 mg/100 g), but it would also identify the need for a simultaneous reduction in total fat. The non-compensatory algorithm makes the system more stringent in categories with complex food matrices like chocolates and cheeses and more permissive for categories with only one limiting nutrient (main potential target for reformulation), which is the case for beverages (main focus: sugar) or salty snacks (main focus sodium). Multi-nutrient systems such as NNPS can therefore help identify opportunities for reformulation across nutrients, avoiding improvement in a single nutrient at the detriment of others.

Another NNPS characteristic is the assessment of the nutrient content per serving, not per 100 g as in many other systems. As previously shown, each reference amount has its strengths and limitations. NP systems based on 100 g would better identify energy-dense foods, and would thereby penalise more heavily products consumed in small serving sizes (e.g., cheese, crackers, nuts, oils), whereas they would be more lenient for products which are not energy-dense and consumed in large portions (e.g., beverages). This explains the differentiation between liquids, solids, and added fats/nuts and seeds categorisation observed in several systems [54]. Meanwhile, nutrient assessment per serving was shown to be similar to assessment per 100 kcal, and was identified as a preferable option in the case of nutrients to encourage [54]. Therefore, the NNPS carries the strengths and limitation of the serving approach, with the additional challenge that regulated serving sizes are not defined everywhere [54]. This explains a limitation of our analysis—similar serving sizes (US RACCs) [32] were used across the five countries—and present a hurdle for the global applicability of the system. At the national level, the system should be applied using either the local regulated serving sizes or serving size derived from national food surveys, i.e., usually consumed. A wider availability of regulated, or at least clearly labeled, portion size information would support the applicability of nutrient profiling per serving. Recent evidence showed that portion sizes across Europe tended to be similar for different food categories [55], and some manufacturers have recently committed to labelling more clear nutritional information per serving (alongside per 100 g) [56]—which would make the use of nutrient profiling systems per serving more user-friendly. 

Currently, only a few countries have established regulated serving sizes despite research showing that such an exercise would be feasible on a national or even regional level. In such a case, the system would highlight further reformulation potential if local serving sizes were higher than the RACC and vice versa. A multi-nutrient system for reformulation is likely to include diverse food categories and hence the hypothesis was that a serving size approach would treat all products with equal strictness, but, in the absence of regulated serving sizes, it can be one of the systems’ limitations, especially when applying to external databases.

There is a recognized need for a shift in the global food supply, to cater for more sustainable dietary choices in terms of health, but also environmental impact [57,58]. This requires sector-wide agreements and close collaboration between governments and the private sector, which are both challenging, with sometimes mixed results [59]. Initiatives exist to regulate marketing [13] and promote consumer education through labelling [9,10], but a limited number of strategies designed to set manufacturing standards exist [20,21,38]. Non-consumer facing campaigns (health by stealth) have not been favoured globally despite reports classifying them among the potentially most cost effective [3,42]. Moreover, it is desirable for more countries to follow best practices and aim to measure the actual impact of the potential reformulations at the diet level [60,61,62,63]. In fact, for reformulation to represent an effective public health strategy, it would need to focus on highly consumed foods and/or most common sources of specific nutrients. For example, pizza, the second most commonly consumed food among US children and teens, has been used previously for such analyses [64]. As highlighted previously, reformulation following a similar system can result in varying nutritional impact between countries [3]. The results of such modeling analyses could also inform the optimal level of specific nutrient criteria to ensure their public health relevance.

For any food level policy to be properly designed and validated, there is a need for easier access to high-quality branded food composition data. Retailers are an important stakeholder in the provision of food label data, while manufacturers have access to important data (e.g., added sugar content) that are most relevant for public health research. Public-private partnerships between retailers, governments, the academic sector and manufacturers have the potential to support the creation of such databases and should be encouraged as a potential platform for better characterisation of the food landscape and its impact on diet and health.

## 5. Conclusions 

This is the first testing of a category-specific nutrient profiling system for reformulation using national food composition datasets from three continents. Overall, the system had acceptable inter-rater agreement for product classification in all datasets. A third of the products met all nutrient criteria, i.e., they obtained an NNPS Pass status, with the system showing usefulness as a tool to encourage reformulation.

These findings are first and foremost of interest to the food manufacturers, retailers, public health or nutritional scientists and policy makers focusing on food policies, and demonstrate the difficulty to design one system relevant to encourage reformulation across multiple countries. Our findings reiterate the need and importance of creating public, (freely) available branded food composition datasets with high granularity and data completeness. Such datasets would advance the capacity to design, validate and measure the potential impact of any reformulation strategy. 

## Figures and Tables

**Table 1:** Description of the granularity and data quality of the datasets used.

Dataset	Number of Items Categorised and Scored (Amenable to Reformulation)		Number of Categories with Products Available for Analysis		Data Imputed & Calculated (*n* (%))		
	*n*	% ^†^	≥1 Item	≥10 Items	SFA	Added Sugars	Sodium
UK	1527	45%	31	23	0 (0%)	1527 (100%)	0 (0%)
France	913	68%	29	20	0 (0%)	0 (0%)	0 (0%)
US	3135	41%	34	29	0 (0%)	0 (0%)	0 (0%)
Brazil	987	50%	30	14	0 (0%)	664 (67%)	0 (0%)
China	621	34%	27	16	247 (60%)	621 (100%)	0 (0%)

† % of items categorised and scored as a function of the total number of food products in the national datasets; SFA: Saturated Fatty Acids.

**Table 2:** Inter-user agreement scores (%) for categorisation of foods from the five different databases, based on correct allocation to categories (*n* = 1719).

		Food Composition Tables				
		France	UK	US	Brazil	China
Product Sample *n* (%)		267 (20%)	350 (10%)	702 (9%)	200 (10%)	200 (11%)
Products classified (%) to the same category by all three reviewers		68%	64%	51%	88%	64%
Kappa statistics	1–2 ^†^	0.71	0.73	0.64	0.72	0.72
	2–3 ^†^	0.84	0.71	0.68	0.81	0.70
	1–3 ^†^	0.76	0.68	0.62	0.87	0.68

^†^ 1, 2 and 3 refer to the three research centres categorising the products: UK, France, Switzerland, respectively.

**Table 3:** Products *n* (%) not meeting the Nestlé Nutritional Profiling System criteria (NNPS Fail) due to different nutrient targets per country.

		Overall *n* (%)		France *n* (%)		UK *n* (%)		China *n* (%)		Brazil *n* (%)		US *n* (%)		*p*-Value (χ^2^) ^†^
Number of nutrient criteria not met *	one criterion only	1797	40%	194	31%	341	33%	170	43%	232	39%	860	45%	0.09
	two criteria	1244	27%	194	31%	334	33%	75	19%	156	26%	485	25%	0.34
	three criteria	880	19%	147	24%	213	21%	91	23%	131	22%	298	16%	0.58
	more than three criteria	624	14%	84	14%	132	13%	60	15%	71	12%	277	14%	0.94
Products that do not meet the criterion - ^#^	energy	1347	19%	188	21%	305	20%	160	26%	200	20%	494	16%	0.72
	total fat	2746	38%	383	42%	720	47%	192	31%	389	39%	1062	34%	0.06
	saturated fat	2116	29%	399	44%	564	37%	168	27%	276	28%	709	23%	0.002
	added sugar	1119	16%	149	16%	278	18%	103	17%	129	13%	460	15%	0.66
	sodium	1707	24%	202	22%	335	22%	117	19%	163	17%	890	28%	0.29
	protein *^#^*	498	14%	28	4%	171	25%	55	24%	51	9%	193	13%	0.000
	calcium *^#^*	216	43%	37	41%	19	22%	38	46%	30	57%	92	49%	0.000
	fibre *^#^*	36	38%	-	-		0%	6	21%	1	25%	29	48%	0.06

† chi-squares between countries. * % calculated on the total number of products that do not meet the criteria. ^#^ % calculated based on the total number of product to which the criteria apply (i.e., all products for energy, total fat, saturated fat, added sugars and sodium, and specific categories only for protein, calcium and fibre).

**Table 4:** Impact of reaching the Nestlé Nutritional Profiling System (NNPS) threshold in all foods amenable to reformulation on nutrients to limit, for key categories (with *n* > 5).

Nutrient	Key Categories	*n*	% Weighted Reduction (Weight Average All Countries) ^#^	Min%–Max% Reduction	Absolute Weighted Reduction (Weight Average)
Energy	Cakes, cookies & desserts	700	−15%	11% (BR)–21% (FR)	50.4 kcal/serving
	Malt-based beverages	8	−13%	0% (US)–81% (CN)	110.7 kcal/serving
	Culinary sauces as accessory	50	−11%	0% (BR)–69% (CN)	30 kcal/serving
	Culinary sauces	46	−11%	0% (BR)–69% (CN)	35.9 kcal/serving
	Dressings	259	−10%	0% (BR)–13% (CN)	12.5 kcal/serving
Total fat	Dressings	259	−30%	19% (BR)–33% (FR/CN/UK)	3.8 g/serving
	Culinary sauces	46	−30%	0% (CN)–46% (UK)	5.9 g/serving
	Culinary sauces as accessory	50	−27%	15% (UK)–88% (CN)	4.8 g/serving
	Cold cuts & spreads	260	−26%	22% (CN)–29% (FR)	4.9 g/serving
	Confectionery bars	97	−19%	7% (BR)–53% (CN)	2.6 g/serving
Saturated fats	Dressings	259	−45%	35% (US)–51% (BR/FR)	1.8 g/serving
	Ice creams	81	−28%	22% (CN)–40% (FR)	2.6 g/serving
	Cold cuts & spreads	260	−28%	21% (CN)–35% (BR)	1.8 g/serving
	Culinary sauces as accessory	50	−27%	0% (CN)–73% (BR)	2.4 g/serving
	Culinary sauces	46	−24%	0% (CN)–37% (UK)	1.9 g/serving
Added sugar	Juice-based beverages	165	−29%	23% (BR)–35% (US)	6.3 g/serving
	Chocolate	99	−25%	21% (BR/US)–45% (CN)	4.9 g/serving
	Water ice creams	10	−25%	19% (US)–43% (FR)	6 g/serving
	Culinary sauces as accessory	50	−19%	0% (BR/FR/CN)–38% (UK)	2.5 g/serving
	Cakes, cookies & desserts	700	−18%	15% (US)–24% (CN)	4.3 g/serving
Sodium	Cold cuts & spreads	260	−48%	43% (CN/FR)–53% (UK)	365.7 mg/serving
	Cold sauces	89	−34%	20% (UK)–64% (CN)	230.6 mg/serving
	Cereal-based foods	69	−30%	0% (FR/CN)–36% (US)	145 mg/serving
	Culinary sauces as accessory	50	−21%	0% (BR)–64% (CN)	258.9 mg/serving
	Culinary sauces	46	−16%	0% (CN)–34% (FR)	66.5 mg/serving

^#^ Weighted reduction achieved across the whole supply when target nutrients are amended to the required NNPS threshold in foods amenable to reformulation (FARs). CN: China, UK: United Kingdom, US: United States of Americas, BR: Brazil, FR: France.

**Table 5:** Impact of reaching the Nestlé Nutritional Profiling System threshold in all foods amenable to reformulation on nutrients to promote, for key categories.

Nutrient	Key Categories	*n*	% Weighted Increase (Weight Average All Countries) ^#^	Min%–Max% Increase	Absolute Weighted Increase (Weight Average)
Protein	Cereal-based foods	69	39%	1% (US)–274% (CN)	5.4 g/serving
	Center of plates	1473	25%	1% (US/UK)–271% (CN)	1.8 g/serving
	Milk-based beverages	194	32%	0% (UK/US/FR)–124% (CN)	0.2 g/serving
	Small meals	506	1%	0% (US)–21% (CN)	0.3 g/serving
Calcium	Cereal-based foods	69	1006%	0% (UK)–4212% (BR)	160.5 mg/serving
	Milk-based beverages	194	279%	5% (UK)–1049% (CN)	28.7 mg/serving
	Yoghurts & fresh cheeses	144	38%	13% (US)–72% (UKR)	25.4 mg/serving
	Dairy desserts	93	24%	1% (FR)–48% (BR)	9.3 mg/serving
Fibre	Cereal-based foods	69	86%	0% (FR)–542% (CN)	0.5 g/serving

^#^ Weighted increase achieved across the whole supply when target nutrients are amended to the required NNPS threshold in foods amenable to reformulation (FARs). CN: China, UK: United Kingdom, US: United States of America, BR: Brazil, FR: France.

## Supplementary Materials

The following are available online at www.mdpi.com/2072-6643/9/4/406/s1, Table S1—Distribution of food items (*n*, %) from UK, Brazil, China, France and the US across NNPS categories; Table S2—NNPS pass rate (%) per nutrient per category for all products in (a) the UK, (b) France, (c) the US, (d) Brazil, (e) China; Table S3—Average nutrient content comparison between NNPS pass/fail products in (a) the UK, (b) France, (c) the US, (d) Brazil, (e) China; Table S4—Minimum reformulation (%) required to reach NNPS threshold in relevant products for each category in (a) the UK, (b) France, (c) the US, (d) Brazil, (e) China; Table S5—Changes to the nutrient composition of all products in scope (%) when minimum reformulation required to reach NNPS threshold is applied (a) the UK, (b) France, (c) US, (d) Brazil, (e) China; Figure S1—Added sugar algorithm for UK products.
