# Supplementary material for: Testing the Capacity of a Multi-Nutrient Profiling System to Guide Food and Beverage Reformulation: Results from Five National Food Composition Databases
Source: Nutrients. 2017 Apr 21;9(4):406. doi: 10.3390/nu9040406 (PMC5409745; doi:10.3390/nu9040406)
Supplement: Supplementary file 1 [file nutrients-09-00406-s001.docx]

**SUPPLEMENTARY FIGURES**

**FIGURE S1 - Added sugar algorithm for UK products**

**SUPPLEMENTARY TABLES**

**TABLE S1 – Distribution of food items (n, %) from UK, Brazil, China, France and the US across NNPS categories**

**TABLE S2– NNPS pass rate (%) per nutrient per category for all products in**

a - UK

b- France

c - US

d - Brazil

e - China

**TABLE S3 – Average nutrient content comparison between NNPS pass/fail products**

a - UK

b- France

c - US

d - Brazil

e – China

**TABLE S4 – Minimum reformulation (%) required to reach NNPS threshold in relevant products for each category**

a - UK

b- France

c - US

d - Brazil

e – China

**TABLE S5 – Changes to the nutrient composition of all products in scope (%) when minimum reformulation required to reach NNPS threshold is applied**

a - UK

b- France

c - US

d - Brazil

e – China

Table S1. Distribution of food items (n, %) in scope from UK, Brazil, China, France and the US across NNPS categories

| **NNPS Categories** | | **UK** | | **Brazil** | | **China** | | **France** | | **US** | |
| --- | --- | --- | --- | --- | --- | --- | --- | --- | --- | --- | --- |
|  |  | **nn** | **%** | **nn** | **%** | **nn** | **%** | **nn** | **%** | **nn** | **%** |
| **1** | **Milk based breakfast beverage** | 3 | 0% | 0 | - | 0 | - | 0 | - | 1 | 0% |
| **2** | **Cereal based foods** | 0 | - | 4 | 0% | 7 | 0% | 1 | 0% | 57 | 0% |
| **3** | **Complete meals** | 78 | 31% | 37 | 38% | 12 | 8% | 23 | 3% | 329 | 42% |
| **4** | **Meat/fish/replacers as centre of plate** | 372 | 32% | 392 | 39% | 110 | 19% | 82 | 9% | 517 | 45% |
| **5** | **Small meals** | 95 | 4% | 39 | 18% | 16 | 31% | 55 | 6% | 301 | 16% |
| **6** | **Side dish** | 264 | 63% | 143 | 81% | 95 | 74% | 65 | 7% | 365 | 73% |
| **7** | **Asian noodles as main dish** | 3 | 0% | 5 | 80% | 7 | 43% | 0 | - | 24 | 54% |
| **8** | **Pizza as centre of plate** | 10 | 0% | 8 | 0% | 0 | - | 9 | 1% | 57 | 25% |
| **9** | **Soups** | 24 | 0% | 33 | 6% | 5 | 0% | 15 | 2% | 147 | 31% |
| **10** | **Cold cuts & spreads** | 57 | 5% | 25 | 8% | 25 | 16% | 60 | 7% | 93 | 3% |
| **11** | **Salty & savoury snacks** | 28 | 29% | 13 | 31% | 19 | 58% | 13 | 1% | 102 | 73% |
| **12** | **Cheeses** | 36 | 11% | 22 | 36% | 7 | 14% | 102 | 11% | 52 | 35% |
| **13** | **Yoghurts & fresh cheeses** | 33 | 33% | 20 | 15% | 10 | 10% | 49 | 5% | 32 | 22% |
| **14** | **Dairy desserts** | 25 | 52% | 5 | 0% | 0 | - | 24 | 3% | 39 | 21% |
| **15** | **Ice creams** | 16 | 13% | 2 | 0% | 12 | 0% | 6 | 1% | 45 | 24% |
| **16** | **Low fat ice creams** | 3 | 0% | 1 | 0% | 0 | - | 0 | - | 19 | 37% |
| **17** | **Water ices & sorbets** | 0 | - | 3 | 0% | 0 | - | 1 | - | 6 | 50% |
| **18** | **Enriched beverages** | 0 | - | 0 | - | 0 | - | 0 | - | 0 | - |
| **19** | **Culinary Sauces** | 19 | 0% | 4 | 25% | 1 | 100% | 11 | 1% | 11 | 18% |
| **20** | **Milk based beverages as snack** | 39 | 77% | 33 | 33% | 49 | 20% | 16 | 2% | 57 | 54% |
| **21** | **Malt based beverages as snack** | 4 | 50% | 0 | - | 1 | 0% | 1 | 0% | 2 | 0% |
| **22** | **Cereal based beverages** | 0 | - | 0 | - | 0 | - | 0 | - | 4 | 0% |
| **23** | **Confectionary bars (non-chocolate based)** | 9 | 0% | 8 | 25% | 5 | 0% | 19 | 2% | 56 | 9% |
| **24** | **Chocolate** | 17 | 0% | 9 | 0% | 3 | 0% | 17 | 2% | 53 | 8% |
| **25** | **Juice based beverages** | 30 | 63% | 29 | 72% | 17 | 41% | 28 | 3% | 61 | 51% |
| **26** | **Cakes, cookies & desserts** | 139 | 17% | 86 | 24% | 80 | 33% | 89 | 10% | 306 | 25% |
| **27** | **Beverages** | 32 | 84% | 17 | 65% | 39 | 49% | 80 | 9% | 122 | 59% |
| **28** | **Sugar confectionery** | 43 | 23% | 27 | 44% | 24 | 92% | 20 | 2% | 84 | 56% |
| **29** | **Dairy accessory** | 34 | 41% | 6 | 67% | 5 | 0% | 16 | 2% | 25 | 72% |
| **30** | **Dressings** | 63 | 11% | 6 | 0% | 38 | 26% | 85 | 9% | 67 | 9% |
| **31** | **Mayonnaise** | 3 | 33% | 2 | 0% | 0 | - | 4 | 0% | 8 | 50% |
| **32** | **Cold sauces** | 26 | 35% | 3 | 33% | 20 | 15% | 9 | 1% | 31 | 26% |
| **33** | **Bouillons & seasonings** | 13 | 62% | 3 | 33% | 12 | 75% | 5 | 1% | 16 | 38% |
| **34** | **Culinary sauces as accessory** | 8 | 13% | 2 | 0% | 1 | 0% | 8 | 1% | 31 | 10% |
| **35** | **Creamers** | 1 | 0% | 0 | - | 1 | 100% | 0 | - | 15 | 100% |
| **Products categorised** | | 1,527 |  | 987 |  | 621 |  | 913 |  | 3,135 |  |
| Products not in scope n (%) | | 1,831 |  | 984 |  | 1,184 |  | 434 |  | 4,483 |  |
| Products (Total) | | 3,425 |  | 1,971 |  | 1,805 |  | 1,347 |  | 7,618 |  |

Table S2a. NNPS pass rate (%) per nutrient per category for all UK products in scope

|  | **NNPS Categories** | **n** | **Energy (kcal)** | **Total fat** | **Saturated fat** | **Added sugar** | **Sodium** | **Protein** | **Calcium** | **Fibre** | **Overall** |
| --- | --- | --- | --- | --- | --- | --- | --- | --- | --- | --- | --- |
| *1* | **Milk-based breakfast beverages** |  | 100% | 0% | 0% | 100% | 100% | 100% | 100% | NA | **0%** |
| *2* | **Cereal-based foods** | **3** | - | - | - | - | - | - | - | - | **-** |
| *3* | **Complete meals** | **0** | 95% | 35% | 51% | 97% | 92% | 87% | NA | NA | **31%** |
| *4* | **Centre of plates** | **78** | 91% | 46% | 72% | 99% | 85% | 70% | NA | NA | **32%** |
| *5* | **Small meals** | **372** | 77% | 21% | 63% | 92% | 73% | 54% | NA | NA | **4%** |
| *6* | **Side dish** | **95** | 97% | 74% | 88% | 97% | 89% | NA | NA | NA | **63%** |
| *7* | **Asian noodles as main dish** | **264** | 100% | 33% | 100% | 100% | 33% | NA | NA | NA | **0%** |
| *8* | **Pizza as a centre of plate** | **3** | 20% | 60% | 80% | 100% | 80% | 80% | NA | NA | **0%** |
| *9* | **Soups** | **10** | 79% | 25% | 58% | 25% | 33% | NA | NA | NA | **0%** |
| *10* | **Cold cuts & spreads** | **24** | 89% | 40% | 35% | 100% | 11% | NA | NA | NA | **5%** |
| *11* | **Salty & savoury snacks** | **57** | 100% | 86% | 68% | 100% | 54% | NA | NA | NA | **29%** |
| *12* | **Cheeses** | **28** | 100% | 22% | 14% | 100% | 86% | 100% | NA | NA | **11%** |
| *13* | **Yoghurts & fresh cheeses** | **36** | 79% | 55% | 52% | 79% | 61% | 79% | 52% | NA | **33%** |
| *14* | **Dairy desserts** | **33** | 80% | 68% | 84% | 72% | 84% | 100% | 84% | NA | **52%** |
| *15* | **Ice creams** | **25** | 56% | 50% | 25% | 38% | 88% | NA | NA | NA | **13%** |
| *16* | **Low-fat ice creams** | **16** | 100% | 100% | 67% | 33% | 100% | NA | NA | NA | **0%** |
| *17* | **Water ices & sorbets** | **3** | - | - | - | - | - | NA | NA | NA | **-** |
| *18* | **Enriched beverages** | **0** | - | - | - | - | - | - | NA | NA | **-** |
| *19* | **Culinary sauces** | **0** | 32% | 16% | 32% | 37% | 53% | NA | NA | NA | **0%** |
| *20* | **Milk-based beverages** | **19** | 69% | 64% | 62% | 67% | 72% | 74% | 67% | NA | **77%** |
| *21* | **Malt-based beverages** | **39** | 50% | 50% | 50% | 100% | 100% | NA | NA | NA | **50%** |
| *22* | **Cereal based beverages** | **4** | - | - | - | - | - | NA | NA | - | **-** |
| *23* | **Confectionary bars** | **0** | 89% | 11% | 33% | 89% | 89% | NA | NA | NA | **0%** |
| *24* | **Chocolate** | **9** | 71% | 53% | 18% | 0% | 100% | NA | NA | NA | **0%** |
| *25* | **Juice-based beverages** | **17** | 100% | 100% | 100% | 70% | 93% | NA | NA | NA | **63%** |
| *26* | **Cakes, cookies & desserts** | **30** | 50% | 65% | 47% | 45% | 72% | NA | NA | NA | **17%** |
| *27* | **Beverages** | **139** | 31% | 38% | 38% | 22% | 34% | NA | NA | NA | **84%** |
| *28* | **Sugar confectionary** | **32** | 53% | 77% | 79% | 30% | 95% | NA | NA | NA | **23%** |
| *29* | **Dairy accessories** | **43** | 74% | 41% | 38% | 88% | 85% | NA | NA | NA | **41%** |
| *30* | **Dressings** | **34** | 37% | 27% | 24% | 87% | 90% | NA | NA | NA | **11%** |
| *31* | **Mayonnaise** | **63** | 33% | 67% | 33% | 100% | 100% | NA | NA | NA | **33%** |
| *32* | **Cold sauces** | **3** | 100% | 100% | 88% | 54% | 62% | NA | NA | NA | **35%** |
| *33* | **Bouillons & seasonings** | **26** | 92% | 92% | 92% | 100% | 69% | NA | NA | NA | **62%** |
| *34* | **Culinary sauces as accessory** | **13** | 88% | 63% | 75% | 38% | 63% | NA | NA | NA | **13%** |
| *35* | **Creamers** | **8** | 0% | 0% | 0% | 100% | 100% | NA | NA | NA | **0%** |

*N/A: Not Applicable*

Table S2b. NNPS pass rate (%) per nutrient per category for all French products in scope

|  | **NNPS Categories** | **n** | **Energy (kcal)** | **Total fat** | **Saturated fat** | **Added sugar** | **Sodium** | **Protein** | **Calcium** | **Fibre** | **Overall** |
| --- | --- | --- | --- | --- | --- | --- | --- | --- | --- | --- | --- |
| *1* | **Milk-based breakfast beverages** | **0** | - | - | - | - | - | - | - | N/A | **-** |
| *2* | **Cereal-based foods** | **1** | 0% | 100% | 100% | 100% | 100% | 0% | 0% | 100% | **0%** |
| *3* | **Complete meals** | **23** | 100% | 52% | 61% | 100% | 100% | 100% | N/A | N/A | **48%** |
| *4* | **Centre of plates** | **82** | 100% | 67% | 78% | 100% | 90% | 92% | N/A | N/A | **37%** |
| *5* | **Small meals** | **55** | 65% | 40% | 65% | 98% | 44% | 93% | N/A | N/A | **15%** |
| *6* | **Side dish** | **65** | 100% | 86% | 98% | 100% | 89% | N/A | N/A | N/A | **78%** |
| *7* | **Asian noodles as main dish** | **0** | - | - | - | - | - | N/A | N/A | N/A | **-** |
| *8* | **Pizza as a centre of plate** | **9** | 100% | 89% | 100% | 100% | 89% | 100% | N/A | N/A | **78%** |
| *9* | **Soups** | **15** | 100% | 93% | 73% | 93% | 67% | N/A | N/A | N/A | **47%** |
| *10* | **Cold cuts & spreads** | **60** | 88% | 30% | 42% | 100% | 13% | N/A | N/A | N/A | **2%** |
| *11* | **Salty & savoury snacks** | **13** | 100% | 92% | 62% | 100% | 92% | N/A | N/A | N/A | **54%** |
| *12* | **Cheeses** | **102** | 100% | 28% | 19% | 100% | 93% | 93% | N/A | N/A | **19%** |
| *13* | **Yoghurts & fresh cheeses** | **49** | 67% | 69% | 69% | 67% | 100% | 96% | 47% | N/A | **33%** |
| *14* | **Dairy desserts** | **24** | 79% | 79% | 79% | 17% | 100% | 100% | 83% | N/A | **17%** |
| *15* | **Ice creams** | **6** | 50% | 50% | 0% | 0% | 100% | N/A | N/A | N/A | **0%** |
| *16* | **Low-fat ice creams** | **0** | N/A | N/A | N/A | N/A | N/A | N/A | N/A | N/A | **N/A** |
| *17* | **Water ices & sorbets** | **1** | 0% | 100% | 100% | 0% | 100% | N/A | N/A | N/A | **0%** |
| *18* | **Enriched beverages** | **0** | - | - | - | - | - | - | - | N/A | **-** |
| *19* | **Culinary sauces** | **11** | 91% | 36% | 45% | 91% | 9% | N/A | N/A | N/A | **0%** |
| *20* | **Milk-based beverages** | **16** | 94% | 94% | 94% | 63% | 100% | 100% | 63% | N/A | **38%** |
| *21* | **Malt-based beverages** | **1** | 100% | 100% | 100% | 100% | 100% | N/A | N/A | N/A | **100%** |
| *22* | **Cereal based beverages** | **0** | - | - | - | - | - | N/A | N/A | - | **-** |
| *23* | **Confectionary bars** | **19** | 100% | 68% | 63% | 74% | 74% | N/A | N/A | N/A | **37%** |
| *24* | **Chocolate** | **17** | 41% | 41% | 18% | 12% | 100% | N/A | N/A | N/A | **0%** |
| *25* | **Juice-based beverages** | **28** | 100% | 100% | 100% | 71% | 86% | N/A | N/A | N/A | **57%** |
| *26* | **Cakes, cookies & desserts** | **89** | 40% | 56% | 34% | 52% | 66% | N/A | N/A | N/A | **12%** |
| *27* | **Beverages** | **80** | 94% | 100% | 100% | 83% | 90% | N/A | N/A | N/A | **73%** |
| *28* | **Sugar confectionary** | **20** | 90% | 95% | 100% | 75% | 100% | N/A | N/A | N/A | **75%** |
| *29* | **Dairy accessories** | **16** | 94% | 81% | 75% | 94% | 100% | N/A | N/A | N/A | **75%** |
| *30* | **Dressings** | **85** | 38% | 16% | 7% | 100% | 96% | N/A | N/A | N/A | **1%** |
| *31* | **Mayonnaise** | **4** | 25% | 100% | 25% | 100% | 100% | N/A | N/A | N/A | **25%** |
| *32* | **Cold sauces** | **9** | 78% | 56% | 44% | 78% | 22% | N/A | N/A | N/A | **0%** |
| *33* | **Bouillons & seasonings** | **5** | 100% | 100% | 100% | 100% | 40% | N/A | N/A | N/A | **40%** |
| *34* | **Culinary sauces as accessory** | **8** | 75% | 38% | 50% | 100% | 75% | N/A | N/A | N/A | **38%** |
| *35* | **Creamers** | **0** | - | - | - | - | - | N/A | N/A | N/A | **-** |

*N/A: Not Applicable*

Table S2c. NNPS pass rate (%) per nutrient per category for all US products in scope

|  | **NNPS Categories** | **n** | **Energy (kcal)** | **Total fat** | **Saturated fat** | **Added sugar** | **Sodium** | **Protein** | **Calcium** | **Fibre** | **Overall** |
| --- | --- | --- | --- | --- | --- | --- | --- | --- | --- | --- | --- |
| *1* | **Milk-based breakfast beverages** | **1** | 100% | 100% | 100% | 0% | 100% | 100% | 100% | NA | **0%** |
| *2* | **Cereal-based foods** | **57** | 49% | 65% | 89% | 96% | 16% | 39% | 12% | 54% | **0%** |
| *3* | **Complete meals** | **329** | 100% | 63% | 83% | 100% | 90% | 81% | NA | NA | **42%** |
| *4* | **Centre of plates** | **517** | 98% | 60% | 85% | 99% | 79% | 90% | NA | NA | **45%** |
| *5* | **Small meals** | **301** | 73% | 32% | 78% | 98% | 43% | 92% | NA | NA | **16%** |
| *6* | **Side dish** | **365** | 99% | 85% | 94% | 96% | 87% | NA | NA | NA | **73%** |
| *7* | **Asian noodles as main dish** | **24** | 96% | 63% | 100% | 100% | 88% | NA | NA | NA | **54%** |
| *8* | **Pizza as a centre of plate** | **57** | 81% | 54% | 70% | 100% | 40% | 96% | NA | NA | **25%** |
| *9* | **Soups** | **147** | 89% | 70% | 58% | 93% | 59% | NA | NA | NA | **31%** |
| *10* | **Cold cuts & spreads** | **93** | 86% | 48% | 53% | 94% | 16% | NA | NA | NA | **3%** |
| *11* | **Salty & savoury snacks** | **102** | 98% | 92% | 96% | 91% | 92% | NA | NA | NA | **73%** |
| *12* | **Cheeses** | **52** | 100% | 48% | 46% | 100% | 85% | 96% | NA | NA | **35%** |
| *13* | **Yoghurts & fresh cheeses** | **32** | 56% | 81% | 84% | 69% | 88% | 97% | 53% | NA | **22%** |
| *14* | **Dairy desserts** | **39** | 85% | 82% | 82% | 44% | 82% | 87% | 56% | NA | **21%** |
| *15* | **Ice creams** | **45** | 56% | 56% | 38% | 36% | 93% | NA | NA | NA | **24%** |
| *16* | **Low-fat ice creams** | **19** | 84% | 89% | 84% | 47% | 84% | NA | NA | NA | **37%** |
| *17* | **Water ices & sorbets** | **6** | 50% | 100% | 83% | 50% | 100% | NA | NA | NA | **50%** |
| *18* | **Enriched beverages** | **0** | - | - | - | - | - | - | - | NA | **-** |
| *19* | **Culinary sauces** | **11** | 82% | 55% | 73% | 100% | 36% | NA | NA | NA | **18%** |
| *20* | **Milk-based beverages** | **57** | 82% | 84% | 84% | 65% | 95% | 84% | 82% | NA | **54%** |
| *21* | **Malt-based beverages** | **2** | 100% | 50% | 0% | 50% | 100% | NA | NA | NA | **0%** |
| *22* | **Cereal based beverages** | **4** | 50% | 100% | 100% | 0% | 100% | NA | NA | 25% | **0%** |
| *23* | **Confectionary bars** | **56** | 91% | 52% | 59% | 75% | 68% | NA | NA | NA | **9%** |
| *24* | **Chocolate** | **53** | 66% | 64% | 40% | 26% | 91% | NA | NA | NA | **8%** |
| *25* | **Juice-based beverages** | **61** | 95% | 97% | 97% | 64% | 84% | NA | NA | NA | **51%** |
| *26* | **Cakes, cookies & desserts** | **306** | 49% | 60% | 62% | 58% | 50% | NA | NA | NA | **25%** |
| *27* | **Beverages** | **122** | 85% | 99% | 98% | 66% | 93% | NA | NA | NA | **59%** |
| *28* | **Sugar confectionary** | **84** | 77% | 94% | 85% | 69% | 100% | NA | NA | NA | **56%** |
| *29* | **Dairy accessories** | **25** | 96% | 84% | 72% | 96% | 100% | NA | NA | NA | **72%** |
| *30* | **Dressings** | **67** | 55% | 39% | 34% | 76% | 67% | NA | NA | NA | **9%** |
| *31* | **Mayonnaise** | **8** | 88% | 100% | 88% | 88% | 75% | NA | NA | NA | **50%** |
| *32* | **Cold sauces** | **31** | 97% | 84% | 77% | 77% | 45% | NA | NA | NA | **26%** |
| *33* | **Bouillons & seasonings** | **16** | 81% | 81% | 88% | 88% | 69% | NA | NA | NA | **38%** |
| *34* | **Culinary sauces as accessory** | **31** | 71% | 52% | 55% | 68% | 45% | NA | NA | NA | **10%** |
| *35* | **Creamers** | **15** | 100% | 100% | 100% | 100% | 100% | NA | NA | NA | **100%** |

*NA: Not Applicable*

Table S2d. NNPS pass rate (%) per nutrient per category for all Brazilian products in scope

|  | **NNPS Categories** | **n** | **Energy (kcal)** | **Total fat** | **Saturated fat** | **Added sugar** | **Sodium** | **Protein** | **Calcium** | **Fibre** | **Overall** |
| --- | --- | --- | --- | --- | --- | --- | --- | --- | --- | --- | --- |
| *1* | **Milk-based breakfast beverages** | **0** | - | - | - | - | - | - | - | NA | **-** |
| *2* | **Cereal-based foods** | **4** | 75% | 50% | 100% | 100% | 50% | 0% | 0% | 75% | **0%** |
| *3* | **Complete meals** | **37** | 100% | 46% | 59% | 100% | 78% | 95% | NA | NA | **38%** |
| *4* | **Centre of plates** | **392** | 78% | 44% | 71% | 100% | 89% | 91% | NA | NA | **39%** |
| *5* | **Small meals** | **39** | 64% | 38% | 69% | 97% | 54% | 90% | NA | NA | **18%** |
| *6* | **Side dish** | **143** | 99% | 89% | 94% | 97% | 96% | NA | NA | NA | **81%** |
| *7* | **Asian noodles as main dish** | **5** | 80% | 80% | 100% | 100% | 100% | NA | NA | NA | **80%** |
| *8* | **Pizza as a centre of plate** | **8** | 100% | 75% | 38% | 100% | 13% | 100% | NA | NA | **0%** |
| *9* | **Soups** | **33** | 82% | 79% | 67% | 70% | 55% | NA | NA | NA | **6%** |
| *10* | **Cold cuts & spreads** | **25** | 80% | 40% | 36% | 96% | 16% | NA | NA | NA | **8%** |
| *11* | **Salty & savoury snacks** | **13** | 100% | 92% | 100% | 46% | 92% | NA | NA | NA | **31%** |
| *12* | **Cheeses** | **22** | 100% | 55% | 50% | 100% | 77% | 100% | NA | NA | **36%** |
| *13* | **Yoghurts & fresh cheeses** | **20** | 75% | 30% | 30% | 80% | 85% | 95% | 50% | NA | **15%** |
| *14* | **Dairy desserts** | **5** | 80% | 80% | 80% | 40% | 80% | 0% | 0% | NA | **0%** |
| *15* | **Ice creams** | **2** | 100% | 50% | 0% | 50% | 100% | NA | NA | NA | **0%** |
| *16* | **Low-fat ice creams** | **1** | 100% | 100% | 100% | 0% | 100% | NA | NA | NA | **0%** |
| *17* | **Water ices & sorbets** | **3** | 67% | 100% | 100% | 0% | 100% | NA | NA | NA | **0%** |
| *18* | **Enriched beverages** | **0** | - | - | - | - | - | - | NA | NA | **-** |
| *19* | **Culinary sauces** | **4** | 75% | 75% | 75% | 100% | 50% | NA | NA | NA | **25%** |
| *20* | **Milk-based beverages** | **33** | 67% | 64% | 64% | 55% | 97% | 58% | 27% | NA | **33%** |
| *21* | **Malt-based beverages** | **0** | - | - | - | - | - | NA | NA | NA | **-** |
| *22* | **Cereal based beverages** | **0** | - | - | - | - | - | NA | NA | NA | **-** |
| *23* | **Confectionary bars** | **8** | 100% | 75% | 88% | 38% | 100% | NA | NA | NA | **25%** |
| *24* | **Chocolate** | **9** | 22% | 11% | 0% | 33% | 100% | NA | NA | NA | **0%** |
| *25* | **Juice-based beverages** | **29** | 97% | 97% | 97% | 76% | 93% | NA | NA | NA | **72%** |
| *26* | **Cakes, cookies & desserts** | **86** | 48% | 79% | 65% | 47% | 79% | NA | NA | NA | **24%** |
| *27* | **Beverages** | **17** | 82% | 94% | 88% | 100% | 76% | NA | NA | NA | **65%** |
| *28* | **Sugar confectionary** | **27** | 81% | 74% | 85% | 63% | 100% | NA | NA | NA | **44%** |
| *29* | **Dairy accessories** | **6** | 100% | 67% | 67% | 100% | 100% | NA | NA | NA | **67%** |
| *30* | **Dressings** | **6** | 50% | 50% | 17% | 100% | 83% | NA | NA | NA | **0%** |
| *31* | **Mayonnaise** | **2** | 100% | 100% | 100% | 0% | 0% | NA | NA | NA | **0%** |
| *32* | **Cold sauces** | **3** | 100% | 100% | 100% | 33% | 33% | NA | NA | NA | **33%** |
| *33* | **Bouillons & seasonings** | **3** | 100% | 100% | 100% | 100% | 33% | NA | NA | NA | **33%** |
| *34* | **Culinary sauces as accessory** | **2** | 100% | 0% | 0% | 100% | 100% | NA | NA | NA | **0%** |
| *35* | **Creamers** | **0** | - | - | - | - | - | NA | NA | NA | **-** |

*N/A: Not Applicable*

Table S2e. NNPS pass rate per nutrient per category for all Chinese products in scope

|  | **NNPS Categories** | **n** | **Energy (kcal)** | **Total fat** | **Saturated fat** | **Added sugar** | **Sodium** | **Protein** | **Calcium** | **Fibre** | **Overall** |
| --- | --- | --- | --- | --- | --- | --- | --- | --- | --- | --- | --- |
| *1* | **Milk-based breakfast beverages** | **0** | - | - | - | - | - | - | - | NA | **-** |
| *2* | **Cereal-based foods** | **7** | 0% | 86% | 100% | 57% | 100% | 0% | 0% | 14% | **0%** |
| *3* | **Complete meals** | **12** | 92% | 8% | 92% | 100% | 92% | 83% | NA | NA | **8%** |
| *4* | **Centre of plates** | **110** | 85% | 52% | 76% | 99% | 57% | 71% | NA | NA | **19%** |
| *5* | **Small meals** | **16** | 81% | 69% | 94% | 94% | 88% | 69% | NA | NA | **31%** |
| *6* | **Side dish** | **95** | 88% | 92% | 96% | 99% | 89% | NA | NA | NA | **74%** |
| *7* | **Asian noodles as main dish** | **7** | 43% | 43% | 57% | 100% | 71% | NA | NA | NA | **43%** |
| *8* | **Pizza as a centre of plate** | **0** | - | - | - | - | - | - | NA | NA | **-** |
| *9* | **Soups** | **5** | 20% | 40% | 100% | 100% | 100% | NA | NA | NA | **0%** |
| *10* | **Cold cuts & spreads** | **25** | 80% | 56% | 64% | 80% | 24% | NA | NA | NA | **16%** |
| *11* | **Salty & savoury snacks** | **19** | 100% | 79% | 79% | 89% | 100% | NA | NA | NA | **58%** |
| *12* | **Cheeses** | **7** | 100% | 71% | 29% | 100% | 86% | 100% | NA | NA | **14%** |
| *13* | **Yoghurts & fresh cheeses** | **10** | 90% | 60% | 50% | 80% | 100% | 90% | 70% | NA | **10%** |
| *14* | **Dairy desserts** | **0** | - | - | - | - | - | - | - | NA | **-** |
| *15* | **Ice creams** | **12** | 100% | 92% | 25% | 0% | 83% | NA | NA | NA | **0%** |
| *16* | **Low-fat ice creams** | **0** | - | - | - | - | - | NA | NA | NA | **-** |
| *17* | **Water ices & sorbets** | **0** | - | - | - | - | - | NA | NA | NA | **-** |
| *18* | **Enriched beverages** | **0** | - | - | - | - | - | - | NA | NA | **-** |
| *19* | **Culinary sauces** | **1** | 100% | 100% | 100% | 100% | 100% | NA | NA | NA | **100%** |
| *20* | **Milk-based beverages** | **49** | 96% | 69% | 67% | 84% | 92% | 84% | 43% | NA | **20%** |
| *21* | **Malt-based beverages** | **1** | 0% | 0% | 0% | 0% | 0% | NA | NA | NA | **0%** |
| *22* | **Cereal based beverages** | **0** | - | - | - | - | - | NA | NA | - | **-** |
| *23* | **Confectionary bars** | **5** | 100% | 0% | 40% | 100% | 100% | NA | NA | NA | **0%** |
| *24* | **Chocolate** | **3** | 0% | 0% | 0% | 0% | 100% | NA | NA | NA | **0%** |
| *25* | **Juice-based beverages** | **17** | 65% | 100% | 100% | 76% | 88% | NA | NA | NA | **41%** |
| *26* | **Cakes, cookies & desserts** | **80** | 45% | 69% | 49% | 50% | 95% | NA | NA | NA | **33%** |
| *27* | **Beverages** | **39** | 59% | 97% | 92% | 69% | 95% | NA | NA | NA | **49%** |
| *28* | **Sugar confectionary** | **24** | 100% | 96% | 100% | 96% | 100% | NA | NA | NA | **92%** |
| *29* | **Dairy accessories** | **5** | 0% | 20% | 20% | 0% | 100% | NA | NA | NA | **0%** |
| *30* | **Dressings** | **38** | 32% | 32% | 29% | 100% | 97% | NA | NA | NA | **26%** |
| *31* | **Mayonnaise** | **0** | - | - | - | - | - | NA | NA | NA | **-** |
| *32* | **Cold sauces** | **20** | 85% | 85% | 85% | 90% | 25% | NA | NA | NA | **15%** |
| *33* | **Bouillons & seasonings** | **12** | 100% | 100% | 100% | 100% | 75% | NA | NA | NA | **75%** |
| *34* | **Culinary sauces as accessory** | **1** | 0% | 0% | 100% | 100% | 0% | NA | NA | NA | **0%** |
| *35* | **Creamers** | **1** | 100% | 100% | 100% | 100% | 100% | NA | NA | NA | **100%** |

*N/A: Not Applicable*

**Table S3a - Average nutrient content comparison between NNPS pass/fail products in the UK (per 100g)**

| **NNPS Categories** | | **NNPS** | **n** | **Energy** | Total fat | Sat. fat | Added sugar | Sodium | Protein | Calcium | Fibre |
| --- | --- | --- | --- | --- | --- | --- | --- | --- | --- | --- | --- |
| *1* | **Milk-based breakfast beverages** | **Pass** | 0 | - | - | - | - | - | - | - | - |
|  |  | **Fail** | 3 | 72.3 | 4.7 | 3.1 | 0.0 | 39.0 | 3.5 | 129.0 | 0.0 |
| *2* | **Cereal-based foods** | **Pass** | 0 | - | - | - | - | - | - | - | - |
|  |  | **Fail** | 0 | - | - | - | - | - | - | - | - |
| *3* | **Complete meals** | **Pass** | 24 | 124.3 | 3.1 | 1.0 | 1.5 | 221.8 | 6.3 | 25.1 | 1.4 |
|  |  | **Fail** | 54 | 158.1 | 8.9 | 3.3 | 1.7 | 323.9 | 7.8 | 51.8 | 1.0 |
| *4* | **Center of plates** | **Pass** | 119 | 154.8 | 7.2 | 2.0 | 0.8 | 305.8 | 17.7 | 43.3 | 0.7 |
|  |  | **Fail** | 253 | 247.0 | 15.7 | 4.8 | 1.2 | 415.4 | 14.7 | 61.6 | 0.7 |
| *5* | **Small meals** | **Pass** | 4 | 162.8 | 6.3 | 1.3 | 1.8 | 301.3 | 12.5 | 49.8 | 1.1 |
|  |  | **Fail** | 91 | 227.8 | 14.9 | 4.9 | 3.0 | 386.2 | 7.1 | 82.3 | 2.1 |
| *6* | **Side dish** | **Pass** | 167 | 150.7 | 4.8 | 1.2 | 1.9 | 240.2 | 4.8 | 61.5 | 2.0 |
|  |  | **Fail** | 97 | 211.5 | 13.8 | 3.8 | 2.6 | 333.7 | 5.4 | 67.1 | 2.0 |
| *7* | **Asian Noodles** | **Pass** | 0 | - | - | - | - | - | - | - | - |
|  |  | **Fail** | 3 | 118.3 | 5.0 | 0.9 | 1.4 | 540.0 | 7.9 | 20.0 | 0.6 |
| *8* | **Pizza as a center of plate** | **Pass** | 0 | - | - | - | - | - | - | - | - |
|  |  | **Fail** | 10 | 235.1 | 9.9 | 3.6 | 2.3 | 343.6 | 10.5 | 179.0 | 1.7 |
| *9* | **Soups** | **Pass** | 0 | - | - | - | - | - | - | - | - |
|  |  | **Fail** | 24 | 60.3 | 3.0 | 0.9 | 2.2 | 389.8 | 2.2 | 20.2 | 0.7 |
| *10* | **Cold cuts & spreads** | **Pass** | 3 | 132.3 | 5.5 | 0.8 | 0.0 | 116.7 | 19.8 | 9.7 | 0.0 |
|  |  | **Fail** | 54 | 274.7 | 21.1 | 7.0 | 0.5 | 1329.1 | 19.7 | 20.8 | 0.2 |
| *11* | **Salty & savoury snacks** | **Pass** | 8 | 423.5 | 17.0 | 4.2 | 2.1 | 669.8 | 11.2 | 85.5 | 6.0 |
|  |  | **Fail** | 20 | 495.7 | 28.7 | 9.8 | 1.6 | 1077.7 | 8.6 | 41.5 | 3.0 |
| *12* | **Cheeses** | **Pass** | 4 | 296.5 | 17.9 | 11.4 | 0.0 | 474.0 | 33.6 | 628.8 | 0.0 |
|  |  | **Fail** | 32 | 371.9 | 30.7 | 19.4 | 0.0 | 756.5 | 23.6 | 614.3 | 0.0 |
| *13* | **Yoghurts & fresh cheeses** | **Pass** | 11 | 63.5 | 1.4 | 0.9 | 1.5 | 55.4 | 5.8 | 132.7 | 0.0 |
|  |  | **Fail** | 22 | 177.0 | 13.1 | 8.5 | 2.0 | 419.0 | 9.7 | 191.6 | 0.0 |
| *14* | **Dairy desserts** | **Pass** | 13 | 101.6 | 2.7 | 1.5 | 6.2 | 54.7 | 3.9 | 119.0 | 0.0 |
|  |  | **Fail** | 12 | 145.6 | 6.2 | 3.3 | 12.2 | 144.7 | 4.4 | 104.8 | 0.2 |
| *15* | **Ice creams** | **Pass** | 2 | 160.5 | 7.5 | 4.6 | 12.7 | 63.5 | 3.3 | 76.0 | 0.0 |
|  |  | **Fail** | 14 | 228.3 | 14.9 | 9.5 | 15.3 | 62.4 | 3.3 | 84.3 | 0.2 |
| *16* | **Low-fat ice creams** | **Pass** | 0 | - | - | - | - | - | - | - | - |
|  |  | **Fail** | 3 | 167.0 | 7.7 | 4.2 | 14.6 | 64.3 | 3.4 | 110.7 | 0.0 |
| *17* | **Water ice creams** | **Pass** | 0 | - | - | - | - | - | - | - | - |
|  |  | **Fail** | 0 | - | - | - | - | - | - | - | - |
| *18* | **Enriched Beverages** | **Pass** | 0 | - | - | - | - | - | - | - | - |
|  |  | **Fail** | 0 | - | - | - | - | - | - | - | - |
| *19* | **Culinary sauces** | **Pass** | 0 | - | - | - | - | - | - | - | - |
|  |  | **Fail** | 19 | 145.2 | 10.5 | 3.6 | 3.6 | 283.8 | 4.2 | 99.2 | 0.6 |
| *20* | **Milk-based beverages** | **Pass** | 30 | 55.5 | 2.3 | 1.4 | 0.6 | 49.7 | 3.5 | 121.0 | 0.0 |
|  |  | **Fail** | 9 | 70.0 | 2.7 | 1.6 | 3.2 | 66.9 | 3.5 | 106.3 | 0.0 |
| *21* | **Malt-based beverages** | **Pass** | 2 | 74.0 | 1.1 | 0.6 | 4.5 | 52.5 | 3.9 | 197.0 | 0.3 |
|  |  | **Fail** | 2 | 91.1 | 3.1 | 2.1 | 2.3 | 69.9 | 4.1 | 173.1 | 0.2 |
| *22* | **Cereal-based beverages** | **Pass** | 0 | - | - | - | - | - | - | - | - |
|  |  | **Fail** | 0 | - | - | - | - | - | - | - | - |
| *23* | **Confectionary bars** | **Pass** | 0 | - | - | - | - | - | - | - | - |
|  |  | **Fail** | 9 | 523.8 | 39.7 | 7.1 | 11.7 | 254.4 | 12.9 | 62.8 | 4.2 |
| *24* | **Chocolate** | **Pass** | 0 | - | - | - | - | - | - | - | - |
|  |  | **Fail** | 17 | 487.8 | 25.0 | 14.3 | 52.2 | 116.4 | 5.8 | 113.5 | 1.1 |
| *25* | **Juice-based beverages** | **Pass** | 19 | 34.7 | 0.1 | 0.0 | 0.0 | 5.1 | 0.3 | 8.0 | 0.0 |
|  |  | **Fail** | 11 | 25.5 | 0.0 | 0.0 | 4.2 | 29.5 | 0.2 | 5.0 | 0.1 |
| *26* | **Cakes, cookies & desserts** | **Pass** | 24 | 337.0 | 12.9 | 4.0 | 15.6 | 264.4 | 6.5 | 80.0 | 1.6 |
|  |  | **Fail** | 115 | 336.8 | 16.1 | 6.5 | 22.8 | 233.8 | 5.0 | 74.4 | 1.3 |
| *27* | **Beverages** | **Pass** | 27 | 8.9 | 0.1 | 0.1 | 0.3 | 7.6 | 0.7 | 10.2 | 0.0 |
|  |  | **Fail** | 5 | 42.0 | 0.0 | 0.0 | 10.8 | 21.4 | 0.0 | 3.8 | 0.0 |
| *28* | **Sugar confectionary** | **Pass** | 10 | 280.7 | 3.8 | 0.8 | 50.7 | 55.8 | 2.8 | 96.3 | 0.8 |
|  |  | **Fail** | 33 | 354.1 | 7.2 | 2.7 | 66.9 | 119.9 | 2.3 | 47.8 | 0.5 |
| *29* | **Dairy accessories** | **Pass** | 14 | 269.7 | 20.2 | 13.3 | 3.7 | 129.0 | 7.9 | 266.6 | 0.0 |
|  |  | **Fail** | 20 | 285.4 | 23.3 | 16.1 | 4.2 | 97.5 | 6.1 | 191.6 | 0.0 |
| *30* | **Dressings** | **Pass** | 7 | 115.6 | 8.1 | 1.9 | 4.3 | 594.7 | 2.6 | 35.9 | 0.9 |
|  |  | **Fail** | 56 | 690.9 | 74.2 | 24.1 | 4.5 | 367.0 | 0.7 | 6.4 | 0.1 |
| *31* | **Mayonnaise** | **Pass** | 1 | 288.0 | 28.1 | 4.2 | 4.6 | -940.0 | 1.0 | 0.0 | 0.0 |
|  |  | **Fail** | 2 | 738.5 | 81.0 | 12.2 | 0.7 | 398.5 | 1.6 | 13.0 | 0.0 |
| *32* | **Cold sauces** | **Pass** | 9 | 128.2 | 5.7 | 1.3 | 1.1 | 230.2 | 4.1 | 86.7 | 0.6 |
|  |  | **Fail** | 17 | 130.6 | 3.7 | 1.3 | 18.2 | 854.6 | 2.4 | 38.8 | 1.2 |
| *33* | **Bouillons & seasonings** | **Pass** | 8 | 211.4 | 8.3 | 0.1 | 2.0 | 1665.3 | 10.4 | 267.5 | 0.2 |
|  |  | **Fail** | 5 | 162.8 | 9.6 | 0.6 | 3.2 | 8249.2 | 8.2 | 51.0 | 0.3 |
| *34* | **Culinary sauces as accessory** | **Pass** | 1 | 22.0 | 0.3 | 0.2 | 4.9 | 110.0 | 0.3 | 29.0 | 0.0 |
|  |  | **Fail** | 7 | 213.1 | 16.8 | 11.1 | 11.6 | 703.0 | 2.2 | 38.6 | 0.4 |
| *35* | **Creamers** | **Pass** | 0 | - | - | - | - | - | - | - | - |
|  |  | **Fail** | 1 | 540.0 | 34.9 | 32.1 | 9.8 | 200.0 | 2.7 | 4.0 | 0.0 |

*Energy in kcal, all nutrients in gram/100g except for sodium and calcium in mg/100g.*

**Table S3b. Average nutrient content comparison between NNPS pass/fail products in France (per 100g)**

| **NNPS Categories** | | **NNPS** | **n** | **Energy (kcal)** | **Total fat** | **Sat. fat** | **Added sugar** | **Sodium** | **Protein** | **Calcium** | **Fibre** |
| --- | --- | --- | --- | --- | --- | --- | --- | --- | --- | --- | --- |
| *1* | **Milk-based breakfast beverages** | Pass | 0 | - | - | - | - | - | - | - | - |
|  |  | Fail | 0 | - | - | - | - | - | - | - | - |
| *2* | **Cereal-based foods** | Pass | 0 | - | - | - | - | - | - | - | - |
|  |  | Fail | 1 | 66 | 1.1 | 0.3 | 0.0 | 4 | 1.5 | 7 | 1.7 |
| *3* | **Complete meals** | Pass | 11 | 127 | 3.9 | 0.9 | 0.4 | 319 | 7.7 | 26 | 1.5 |
|  |  | Fail | 12 | 139 | 7.1 | 2.8 | 0.1 | 365 | 6.9 | 59 | 1.6 |
| *4* | **Center of plates** | Pass | 30 | 144 | 7.3 | 1.9 | 0.1 | 245 | 17.1 | 50 | 0.5 |
|  |  | Fail | 52 | 226 | 15.6 | 5.5 | 0.2 | 618 | 15.4 | 53 | 0.4 |
| *5* | **Small meals** | Pass | 8 | 229 | 8.1 | 2.6 | 1.0 | 426 | 10.1 | 49 | 1.7 |
|  |  | Fail | 47 | 272 | 14.2 | 5.5 | 1.3 | 544 | 10.4 | 81 | 1.7 |
| *6* | **Side dish** | Pass | 51 | 200 | 3.6 | 1.0 | 0.5 | 313 | 5.8 | 37 | 3.3 |
|  |  | Fail | 14 | 179 | 10.7 | 1.8 | 0.2 | 482 | 4.6 | 28 | 2.3 |
| *7* | **Asian Noodles** | Pass | 0 | - | - | - | - | - | - | - | - |
|  |  | Fail | 0 | - | - | - | - | - | - | - | - |
| *8* | **Pizza as a center of plate** | Pass | 7 | 223 | 8.8 | 2.1 | 0.1 | 490 | 8.9 | 149 | 2.4 |
|  |  | Fail | 2 | 240 | 10.0 | 3.8 | 1.1 | 610 | 8.9 | 125 | 2.8 |
| *9* | **Soups** | Pass | 7 | 34 | 0.6 | 0.1 | 0.0 | 210 | 1.3 | 23 | 1.0 |
|  |  | Fail | 8 | 37 | 1.4 | 0.6 | 0.6 | 397 | 2.1 | 23 | 0.8 |
| *10* | **Cold cuts & spreads** | Pass | 1 | 144 | 7.5 | 2.2 | 0.0 | 973 | 19.2 | 38 | 0.0 |
|  |  | Fail | 59 | 270 | 20.9 | 6.6 | 0.1 | 1135 | 17.7 | 29 | 0.2 |
| *11* | **Salty & savoury snacks** | Pass | 7 | 351 | 19.6 | 5.6 | 1.0 | 745 | 6.5 | 42 | 6.1 |
|  |  | Fail | 6 | 466 | 26.4 | 11.7 | 1.0 | 821 | 8.5 | 92 | 3.1 |
| *12* | **Cheeses** | Pass | 19 | 239 | 16.6 | 9.5 | 0.0 | 563 | 18.0 | 376 | 0.1 |
|  |  | Fail | 83 | 341 | 28.3 | 18.1 | 0.0 | 685 | 20.1 | 473 | 0.0 |
| *13* | **Yoghurts & fresh cheeses** | Pass | 16 | 56 | 1.3 | 0.5 | 0.8 | 47 | 5.1 | 128 | 0.1 |
|  |  | Fail | 33 | 99 | 4.0 | 2.4 | 6.1 | 47 | 4.9 | 119 | 0.1 |
| *14* | **Dairy desserts** | Pass | 4 | 90 | 1.9 | 1.2 | 4.9 | 48 | 3.0 | 87 | 1.1 |
|  |  | Fail | 20 | 149 | 5.6 | 3.1 | 14.5 | 60 | 3.9 | 95 | 0.4 |
| *15* | **Ice creams** | Pass | 0 | - | - | - | - | - | - | - | - |
|  |  | Fail | 6 | 247 | 13.3 | 8.5 | 23.2 | 59 | 3.9 | 107 | 1.2 |
| *16* | **Low-fat ice creams** | Pass | 0 | - | - | - | - | - | - | - | - |
|  |  | Fail | 0 | - | - | - | - | - | - | - | - |
| *17* | **Water ice creams** | Pass | 0 | - | - | - | - | - | - | - | - |
|  |  | Fail | 1 | 123 | 1.6 | 0.0 | 26.0 | 13 | 0.8 | 15 | 1.1 |
| *18* | **Enriched Beverages** | Pass | 0 | - | - | - | - | - | - | - | - |
|  |  | Fail | 0 | - | - | - | - | - | - | - | - |
| *19* | **Culinary sauces** | Pass | 0 | - | - | - | - | - | - | - | - |
|  |  | Fail | 11 | 88 | 4.8 | 1.7 | 1.7 | 493 | 1.9 | 45 | 0.8 |
| *20* | **Milk-based beverages** | Pass | 6 | 60 | 1.4 | 0.8 | 1.7 | 50 | 4.1 | 112 | 0.6 |
|  |  | Fail | 10 | 74 | 1.8 | 1.0 | 7.1 | 44 | 3.3 | 99 | 0.3 |
| *21* | **Malt-based beverages** | Pass | 1 | 78 | 1.9 | 0.9 | 4.5 | 61 | 3.8 | 120 | 0.2 |
|  |  | Fail | 0 | - | - | - | - | - | - | - | - |
| *22* | **Cereal-based beverages** | Pass | 0 | - | - | - | - | - | - | - | - |
|  |  | Fail | 0 | - | - | - | - | - | - | - | - |
| *23* | **Confectionary bars** | Pass | 7 | 283 | 2.6 | 0.2 | 1.1 | 32 | 6.4 | 99 | 6.6 |
|  |  | Fail | 12 | 479 | 23.2 | 6.6 | 23.0 | 280 | 12.0 | 133 | 4.6 |
| *24* | **Chocolate** | Pass | 0 | - | - | - | - | - | - | - | - |
|  |  | Fail | 17 | 522 | 30.3 | 16.6 | 42.9 | 86 | 6.7 | 130 | 4.1 |
| *25* | **Juice-based beverages** | Pass | 16 | 45 | 0.1 | 0.0 | 0.0 | 3 | 0.4 | 18 | 0.2 |
|  |  | Fail | 12 | 42 | 0.1 | 0.0 | 5.2 | 55 | 0.5 | 14 | 0.4 |
| *26* | **Cakes, cookies & desserts** | Pass | 11 | 464 | 17.9 | 6.8 | 23.2 | 322 | 7.2 | 105 | 2.8 |
|  |  | Fail | 78 | 361 | 15.5 | 7.1 | 21.5 | 263 | 5.9 | 59 | 2.0 |
| *27* | **Beverages** | Pass | 58 | 2 | 0.0 | 0.0 | 0.1 | 9 | 0.0 | 14 | 0.0 |
|  |  | Fail | 22 | 25 | 0.0 | 0.0 | 5.6 | 39 | 0.0 | 9 | 0.0 |
| *28* | **Sugar confectionary** | Pass | 15 | 259 | 1.7 | 0.4 | 42.9 | 27 | 1.3 | 16 | 1.5 |
|  |  | Fail | 5 | 335 | 4.4 | 0.4 | 63.7 | 31 | 3.1 | 31 | 2.8 |
| *29* | **Dairy accessories** | Pass | 12 | 173 | 14.3 | 9.2 | 2.4 | 59 | 2.9 | 114 | 0.0 |
|  |  | Fail | 4 | 317 | 26.4 | 18.4 | 10.8 | 47 | 3.6 | 134 | 0.0 |
| *30* | **Dressings** | Pass | 1 | 23 | 0.2 | 0.0 | 0.0 | 8 | 0.1 | 7 | 0.0 |
|  |  | Fail | 84 | 694 | 76.6 | 20.9 | 0.0 | 168 | 0.5 | 10 | 0.0 |
| *31* | **Mayonnaise** | Pass | 1 | 344 | 35.2 | 3.4 | 1.0 | 782 | 1.3 | 47 | 0.0 |
|  |  | Fail | 3 | 717 | 77.8 | 16.6 | 0.0 | 428 | 1.5 | 10 | 0.0 |
| *32* | **Cold sauces** | Pass | 0 | - | - | - | - | - | - | - | - |
|  |  | Fail | 9 | 236 | 19.5 | 5.0 | 2.9 | 925 | 2.9 | 63 | 0.9 |
| *33* | **Bouillons & seasonings** | Pass | 2 | 6 | 0.3 | 0.0 | 0.0 | 259 | 0.5 | 1 | 0.0 |
|  |  | Fail | 3 | 20 | 0.3 | 0.0 | 1.0 | 2380 | 2.1 | 13 | 0.0 |
| *34* | **Culinary sauces as accessory** | Pass | 3 | 58 | 5.1 | 2.0 | 0.0 | 177 | 0.6 | 20 | 0.2 |
|  |  | Fail | 5 | 161 | 14.3 | 7.8 | 0.2 | 422 | 2.6 | 69 | 0.3 |
| *35* | **Creamers** | Pass | 0 | - | - | - | - | - | - | - | - |
|  |  | Fail | 0 | - | - | - | - | - | - | - | - |

*Energy in kcal, all nutrients in gram/100g except for sodium and calcium in mg/100g.*

**Table S3c. Average nutrient content comparison between NNPS pass/fail products in the US (per 100g)**

| **NNPS Categories** | | **NNPS** | | **n** | **Energy (kcal)** | **Total fat** | **Sat. fat** | **Added sugar** | **Sodium** | **Protein** | **Calcium** | **Fibre** |
| --- | --- | --- | --- | --- | --- | --- | --- | --- | --- | --- | --- | --- |
| *1* | **Milk-based breakfast beverages** | | Pass | 0 | - | - | - | - | - | - | - | - |
|  |  |  | Fail | 1 | 90 | 1.8 | 1.1 | 5.7 | 89 | 5.5 | 141 | 0.1 |
| *2* | **Cereal-based foods** | | Pass | 0 | - | - | - | - | - | - | - | - |
|  |  |  | Fail | 57 | 89 | 2.3 | 0.8 | 0.9 | 174 | 2.8 | 76 | 1.3 |
| *3* | **Complete meals** | | Pass | 137 | 119 | 3.2 | 0.9 | 0.3 | 283 | 7.8 | 26 | 1.5 |
|  |  |  | Fail | 192 | 150 | 6.4 | 2.0 | 0.2 | 334 | 7.1 | 46 | 1.2 |
| *4* | **Center of plates** | | Pass | 233 | 163 | 6.9 | 1.7 | 0.2 | 426 | 20.5 | 26 | 0.4 |
|  |  |  | Fail | 284 | 211 | 13.2 | 4.0 | 0.8 | 538 | 16.0 | 55 | 0.6 |
| *5* | **Small meals** | | Pass | 47 | 192 | 6.3 | 1.8 | 1.9 | 375 | 10.7 | 102 | 2.0 |
|  |  |  | Fail | 254 | 246 | 12.8 | 4.3 | 1.7 | 527 | 10.7 | 120 | 1.7 |
| *6* | **Side dish** | | Pass | 265 | 182 | 4.3 | 1.0 | 1.8 | 353 | 6.3 | 61 | 2.8 |
|  |  |  | Fail | 100 | 177 | 9.5 | 2.7 | 1.9 | 420 | 6.0 | 84 | 1.9 |
| *7* | **Asian Noodles** | | Pass | 13 | 118 | 2.6 | 0.5 | 0.0 | 317 | 5.9 | 16 | 1.1 |
|  |  |  | Fail | 11 | 178 | 9.0 | 1.5 | 0.8 | 400 | 9.6 | 31 | 1.1 |
| *8* | **Pizza as a center of plate** | | Pass | 14 | 242 | 9.0 | 3.7 | 1.2 | 518 | 11.1 | 167 | 2.2 |
|  |  |  | Fail | 43 | 281 | 12.9 | 5.3 | 1.0 | 612 | 12.2 | 186 | 2.1 |
| *9* | **Soups** | | Pass | 46 | 44 | 1.0 | 0.2 | 0.0 | 250 | 3.2 | 19 | 1.0 |
|  |  |  | Fail | 101 | 64 | 2.4 | 0.9 | 0.2 | 326 | 3.5 | 30 | 0.8 |
| *10* | **Cold cuts & spreads** | | Pass | 3 | 350 | 21.9 | 3.9 | 4.8 | 911 | 29.9 | 104 | 0.1 |
|  |  |  | Fail | 90 | 289 | 19.9 | 5.8 | 1.7 | 1265 | 21.7 | 43 | 0.9 |
| *11* | **Salty & savoury snacks** | | Pass | 74 | 435 | 14.6 | 2.8 | 1.8 | 541 | 8.5 | 70 | 5.6 |
|  |  |  | Fail | 28 | 478 | 23.0 | 6.4 | 6.0 | 662 | 8.5 | 72 | 3.8 |
| *12* | **Cheeses** | | Pass | 18 | 259 | 16.3 | 9.6 | 0.1 | 783 | 22.9 | 695 | 0.2 |
|  |  |  | Fail | 34 | 345 | 27.3 | 16.5 | 0.0 | 786 | 21.1 | 659 | 0.0 |
| *13* | **Yoghurts & fresh cheeses** | | Pass | 7 | 62 | 1.4 | 0.9 | 0.9 | 63 | 4.6 | 162 | 0.1 |
|  |  |  | Fail | 25 | 110 | 3.6 | 2.0 | 4.3 | 197 | 7.4 | 144 | 0.2 |
| *14* | **Dairy desserts** | | Pass | 8 | 52 | 1.8 | 1.0 | 1.9 | 46 | 2.5 | 78 | 0.1 |
|  |  |  | Fail | 31 | 140 | 5.2 | 2.7 | 13.0 | 138 | 3.1 | 75 | 0.4 |
| *15* | **Ice creams** | | Pass | 11 | 120 | 2.2 | 1.1 | 5.2 | 78 | 4.1 | 130 | 1.4 |
|  |  |  | Fail | 34 | 252 | 13.8 | 7.5 | 18.0 | 86 | 4.2 | 120 | 1.6 |
| *16* | **Low-fat ice creams** | | Pass | 7 | 152 | 4.0 | 2.3 | 8.5 | 80 | 4.4 | 139 | 0.6 |
|  |  |  | Fail | 12 | 205 | 7.6 | 4.6 | 15.1 | 90 | 4.9 | 130 | 1.2 |
| *17* | **Water ice creams** | | Pass | 3 | 84 | 0.1 | 0.0 | 12.5 | 5 | 0.8 | 3 | 0.7 |
|  |  |  | Fail | 3 | 126 | 1.3 | 0.7 | 26.7 | 25 | 0.7 | 15 | 0.1 |
| *18* | **Enriched Beverages** | | Pass | 0 | - | - | - | - | - | - | - | - |
|  |  |  | Fail | 0 | - | - | - | - | - | - | - | - |
| *19* | **Culinary sauces** | | Pass | 2 | 48 | 1.3 | 0.2 | 0.9 | 174 | 1.4 | 26 | 1.8 |
|  |  |  | Fail | 9 | 99 | 6.7 | 1.7 | 0.6 | 341 | 3.6 | 27 | 1.6 |
| *20* | **Milk-based beverages** | | Pass | 31 | 54 | 1.8 | 1.0 | 1.4 | 51 | 3.2 | 118 | 0.1 |
|  |  |  | Fail | 26 | 81 | 2.4 | 1.4 | 7.0 | 64 | 2.8 | 125 | 0.5 |
| *21* | **Malt-based beverages** | | Pass | 0 | - | - | - | - | - | - | - | - |
|  |  |  | Fail | 2 | 78 | 2.3 | 1.3 | 3.5 | 81 | 3.8 | 139 | 0.2 |
| *22* | **Cereal-based beverages** | | Pass | 0 | - | - | - | - | - | - | - | - |
|  |  |  | Fail | 4 | 65 | 0.7 | 0.3 | 10.7 | 8 | 0.8 | 18 | 0.3 |
| *23* | **Confectionary bars** | | Pass | 5 | 397 | 12.0 | 1.5 | 19.0 | 195 | 11.7 | 163 | 8.7 |
|  |  |  | Fail | 51 | 466 | 23.6 | 6.1 | 24.7 | 288 | 11.8 | 176 | 5.8 |
| *24* | **Chocolate** | | Pass | 4 | 371 | 17.7 | 7.7 | 16.5 | 33 | 4.9 | 87 | 2.2 |
|  |  |  | Fail | 49 | 500 | 26.7 | 13.4 | 43.3 | 158 | 7.6 | 109 | 3.3 |
| *25* | **Juice-based beverages** | | Pass | 31 | 42 | 0.2 | 0.0 | 0.0 | 6 | 0.4 | 25 | 0.3 |
|  |  |  | Fail | 30 | 49 | 0.2 | 0.1 | 6.1 | 47 | 0.5 | 18 | 0.5 |
| *26* | **Cakes, cookies & desserts** | | Pass | 77 | 379 | 14.3 | 3.7 | 21.3 | 321 | 4.9 | 41 | 2.2 |
|  |  |  | Fail | 229 | 325 | 14.7 | 4.8 | 18.6 | 320 | 5.1 | 64 | 1.9 |
| *27* | **Beverages** | | Pass | 72 | 8 | 0.1 | 0.0 | 0.9 | 7 | 0.1 | 4 | 0.0 |
|  |  |  | Fail | 50 | 39 | 0.3 | 0.1 | 8.0 | 26 | 0.2 | 11 | 0.0 |
| *28* | **Sugar confectionary** | | Pass | 47 | 293 | 4.5 | 1.7 | 39.3 | 77 | 2.0 | 41 | 1.4 |
|  |  |  | Fail | 37 | 344 | 7.6 | 4.4 | 58.9 | 86 | 1.9 | 42 | 1.1 |
| *29* | **Dairy accessories** | | Pass | 18 | 188 | 13.7 | 9.5 | 5.8 | 68 | 3.6 | 125 | 0.0 |
|  |  |  | Fail | 7 | 215 | 17.4 | 11.5 | 7.0 | 67 | 3.9 | 116 | 0.0 |
| *30* | **Dressings** | | Pass | 6 | 150 | 12.0 | 2.5 | 0.2 | 512 | 2.0 | 34 | 0.4 |
|  |  |  | Fail | 61 | 477 | 49.4 | 14.9 | 5.0 | 648 | 1.0 | 22 | 0.3 |
| *31* | **Mayonnaise** | | Pass | 4 | 228 | 20.8 | 4.1 | 1.9 | 577 | 1.2 | 10 | 0.2 |
|  |  |  | Fail | 4 | 310 | 30.5 | 4.8 | 4.3 | 769 | 0.5 | 7 | 0.5 |
| *32* | **Cold sauces** | | Pass | 8 | 120 | 8.5 | 2.4 | 3.0 | 464 | 2.5 | 39 | 2.0 |
|  |  |  | Fail | 23 | 161 | 10.0 | 3.5 | 7.4 | 1282 | 2.3 | 50 | 1.7 |
| *33* | **Bouillons & seasonings** | | Pass | 6 | 33 | 0.3 | 0.1 | 1.7 | 910 | 2.6 | 27 | 0.1 |
|  |  |  | Fail | 10 | 124 | 6.6 | 0.9 | 6.1 | 960 | 4.3 | 58 | 2.1 |
| *34* | **Culinary sauces as accessory** | | Pass | 3 | 144 | 9.5 | 2.1 | 0.8 | 493 | 5.8 | 44 | 1.0 |
|  |  |  | Fail | 28 | 194 | 13.7 | 6.9 | 7.8 | 1073 | 3.6 | 61 | 0.6 |
| *35* | **Creamers** | | Pass | 15 | 266 | 13.1 | 6.5 | 29.1 | 109 | 1.3 | 5 | 0.3 |
|  |  |  | Fail | 0 | - | - | - | - | - | - | - | - |

*Energy in kcal, all nutrients in gram/100g except for sodium and calcium in mg/100g.*

**Table S3d. Average nutrient content comparison between NNPS pass/fail products in Brazil (per 100g)**

| **NNPS Categories** | | **NNPS** | **n** | **Energy (kcal)** | **Total fat** | **Sat. fat** | **Added sugar** | **Sodium** | **Protein** | **Calcium** | **Fibre** |
| --- | --- | --- | --- | --- | --- | --- | --- | --- | --- | --- | --- |
| *1* | **Milk-based breakfast beverages** | Pass | 0 | - | - | - | - | - | - | - | - |
|  |  | Fail | 0 | - | - | - | - | - | - | - | - |
| *2* | **Cereal-based foods** | Pass | 0 | - | - | - | - | - | - | - | - |
|  |  | Fail | 4 | 120.8 | 3.7 | 0.6 | 3.2 | 125.7 | 2.4 | 11.2 | 2.7 |
| *3* | **Complete meals** | Pass | 14 | 119.0 | 3.2 | 0.7 | 0.6 | 129.7 | 8.6 | 25.0 | 1.6 |
|  |  | Fail | 23 | 166.5 | 8.6 | 3.1 | 1.6 | 460.7 | 10.5 | 34.5 | 1.0 |
| *4* | **Center of plates** | Pass | 152 | 178.2 | 6.8 | 1.9 | 0.2 | 106.0 | 26.2 | 22.5 | 0.1 |
|  |  | Fail | 240 | 277.2 | 19.1 | 6.4 | 0.5 | 392.0 | 23.0 | 18.4 | 0.1 |
| *5* | **Small meals** | Pass | 7 | 237.7 | 7.8 | 2.6 | 1.6 | 403.0 | 9.8 | 114.2 | 1.5 |
|  |  | Fail | 32 | 277.6 | 14.3 | 5.9 | 2.4 | 542.4 | 10.6 | 117.2 | 1.7 |
| *6* | **Side dish** | Pass | 116 | 100.6 | 2.9 | 0.6 | 2.0 | 122.1 | 3.1 | 44.5 | 2.4 |
|  |  | Fail | 27 | 242.0 | 12.4 | 5.2 | 9.4 | 244.7 | 7.7 | 74.8 | 1.9 |
| *7* | **Asian Noodles** | Pass | 4 | 189.9 | 7.2 | 2.4 | 1.7 | 429.7 | 5.7 | 94.0 | 1.2 |
|  |  | Fail | 1 | 527.0 | 30.8 | 4.4 | 1.5 | 439.0 | 8.4 | 20.0 | 3.9 |
| *8* | **Pizza as a center of plate** | Pass | 0 | - | - | - | - | - | - | - | - |
|  |  | Fail | 8 | 263.8 | 11.0 | 5.1 | 0.4 | 697.7 | 13.5 | 238.3 | 1.6 |
| *9* | **Soups** | Pass | 2 | 10.6 | 0.0 | 0.0 | 0.4 | 289.6 | 0.5 | 6.5 | 0.2 |
|  |  | Fail | 31 | 65.2 | 1.8 | 0.6 | 1.0 | 290.0 | 5.5 | 12.5 | 0.5 |
| *10* | **Cold cuts & spreads** | Pass | 2 | 260.8 | 17.8 | 6.1 | 0.3 | 468.5 | 22.5 | 20.5 | 0.0 |
|  |  | Fail | 23 | 320.1 | 21.7 | 7.9 | 3.8 | 1264.8 | 25.2 | 16.6 | 0.3 |
| *11* | **Salty & savoury snacks** | Pass | 4 | 459.4 | 18.5 | 3.6 | 1.9 | 736.3 | 8.9 | 31.1 | 5.2 |
|  |  | Fail | 9 | 445.5 | 17.5 | 4.1 | 14.4 | 683.3 | 8.3 | 32.1 | 4.0 |
| *12* | **Cheeses** | Pass | 8 | 260.5 | 16.6 | 10.5 | 1.6 | 619.8 | 23.7 | 660.0 | 0.0 |
|  |  | Fail | 14 | 315.4 | 23.7 | 15.0 | 3.4 | 914.2 | 21.7 | 603.5 | 0.0 |
| *13* | **Yoghurts & fresh cheeses** | Pass | 3 | 69.9 | 1.2 | 0.8 | 5.1 | 69.0 | 7.9 | 167.0 | 0.0 |
|  |  | Fail | 17 | 184.4 | 12.8 | 8.0 | 5.8 | 298.5 | 9.6 | 226.9 | 0.3 |
| *14* | **Dairy desserts** | Pass | 0 | - | - | - | - | - | - | - | - |
|  |  | Fail | 5 | 133.3 | 4.7 | 2.5 | 14.2 | 121.1 | 4.1 | 100.6 | 0.2 |
| *15* | **Ice creams** | Pass | 0 | - | - | - | - | - | - | - | - |
|  |  | Fail | 2 | 198.0 | 11.9 | 6.8 | 13.0 | 63.3 | 3.4 | 124.8 | 2.8 |
| *16* | **Low-fat ice creams** | Pass | 0 | - | - | - | - | - | - | - | - |
|  |  | Fail | 1 | 189.0 | 7.7 | 4.7 | 17.5 | 75.0 | 4.4 | 149.3 | 0.6 |
| *17* | **Water ice creams** | Pass | 0 | - | - | - | - | - | - | - | - |
|  |  | Fail | 3 | 93.6 | 0.2 | 0.0 | 22.7 | 7.5 | 0.1 | 1.7 | 0.2 |
| *18* | **Enriched Beverages** | Pass | 0 | - | - | - | - | - | - | - | - |
|  |  | Fail | 0 | - | - | - | - | - | - | - | - |
| *19* | **Culinary sauces** | Pass | 1 | 102.7 | 1.0 | 0.3 | 0.4 | 5.6 | 2.4 | 44.5 | 1.9 |
|  |  | Fail | 3 | 88.5 | 3.7 | 1.4 | 1.7 | 387.8 | 6.4 | 29.3 | 1.4 |
| *20* | **Milk-based beverages** | Pass | 11 | 55.2 | 2.2 | 1.3 | 5.3 | 41.8 | 3.3 | 117.5 | 0.0 |
|  |  | Fail | 22 | 121.2 | 3.3 | 1.9 | 13.1 | 82.1 | 4.6 | 144.7 | 0.8 |
| *21* | **Malt-based beverages** | Pass | 0 | - | - | - | - | - | - | - | - |
|  |  | Fail | 0 | - | - | - | - | - | - | - | - |
| *22* | **Cereal-based beverages** | Pass | 0 | - | - | - | - | - | - | - | - |
|  |  | Fail | 0 | - | - | - | - | - | - | - | - |
| *23* | **Confectionary bars** | Pass | 2 | 403.5 | 5.4 | 0.6 | 12.4 | 230.5 | 5.6 | 75.0 | 7.2 |
|  |  | Fail | 6 | 425.2 | 19.4 | 2.7 | 43.2 | 185.6 | 9.3 | 299.0 | 3.6 |
| *24* | **Chocolate** | Pass | 0 | - | - | - | - | - | - | - | - |
|  |  | Fail | 9 | 529.1 | 33.5 | 20.2 | 40.3 | 86.9 | 8.5 | 175.2 | 3.9 |
| *25* | **Juice-based beverages** | Pass | 21 | 42.0 | 0.2 | 0.0 | 0.1 | 8.8 | 0.6 | 10.6 | 0.5 |
|  |  | Fail | 8 | 58.4 | 0.5 | 0.1 | 8.4 | 202.6 | 0.7 | 12.0 | 0.7 |
| *26* | **Cakes, cookies & desserts** | Pass | 21 | 329.6 | 13.3 | 3.8 | 19.4 | 237.8 | 5.8 | 65.8 | 1.6 |
|  |  | Fail | 65 | 336.2 | 12.7 | 5.1 | 24.9 | 223.5 | 5.8 | 55.6 | 1.5 |
| *27* | **Beverages** | Pass | 11 | 6.9 | 0.0 | 0.0 | 1.7 | 0.8 | 0.0 | 2.6 | 0.0 |
|  |  | Fail | 6 | 91.0 | 1.3 | 0.4 | 1.1 | 662.3 | 3.8 | 42.5 | 2.8 |
| *28* | **Sugar confectionary** | Pass | 12 | 192.0 | 2.3 | 1.1 | 32.6 | 43.0 | 2.0 | 66.0 | 0.6 |
|  |  | Fail | 15 | 381.3 | 12.5 | 3.9 | 56.5 | 33.8 | 5.2 | 34.8 | 2.9 |
| *29* | **Dairy accessories** | Pass | 4 | 155.4 | 11.6 | 7.2 | 8.1 | 66.5 | 2.8 | 86.2 | 0.0 |
|  |  | Fail | 2 | 292.0 | 30.9 | 19.3 | 2.8 | 34.0 | 2.2 | 69.0 | 0.0 |
| *30* | **Dressings** | Pass | 0 | - | - | - | - | - | - | - | - |
|  |  | Fail | 6 | 471.8 | 52.8 | 25.5 | 0.8 | 655.3 | 0.6 | 18.8 | 0.2 |
| *31* | **Mayonnaise** | Pass | 0 | - | - | - | - | - | - | - | - |
|  |  | Fail | 2 | 208.8 | 16.4 | 2.7 | 11.6 | 854.0 | 0.5 | 9.2 | 0.7 |
| *32* | **Cold sauces** | Pass | 1 | 67.0 | 4.0 | 0.3 | 0.9 | 1135.0 | 4.4 | 58.0 | 3.3 |
|  |  | Fail | 2 | 97.0 | 0.3 | 0.0 | 16.3 | 1114.0 | 1.7 | 18.0 | 0.3 |
| *33* | **Bouillons & seasonings** | Pass | 1 | 53.1 | 0.1 | 0.0 | 2.1 | 3341.0 | 5.2 | 17.0 | 0.8 |
|  |  | Fail | 2 | 53.0 | 0.0 | 0.0 | 2.0 | 5637.0 | 6.3 | 19.0 | 0.8 |
| *34* | **Culinary sauces as accessory** | Pass | 0 | - | - | - | - | - | - | - | - |
|  |  | Fail | 2 | 202.5 | 20.9 | 18.5 | 4.7 | 12.0 | 1.6 | 4.0 | 0.9 |
| *35* | **Creamers** | Pass | 0 | - | - | - | - | - | - | - | - |
|  |  | Fail | 0 | - | - | - | - | - | - | - | - |

*Energy in kcal, all nutrients in gram/100g except for sodium and calcium in mg/100g.*

**Table S3e. Average nutrient content comparison between NNPS pass/fail products in China (per 100g)**

| **NNPS Categories** | | **NNPS** | **n** | **Energy (kcal)** | **Total fat** | **Sat. fat** | **Added sugar** | **Sodium** | **Protein** | **Calcium** | **Fibre** |
| --- | --- | --- | --- | --- | --- | --- | --- | --- | --- | --- | --- |
| *1* | **Milk-based breakfast beverages** | Pass | 0 | - | - | - | - | - | - | - | - |
|  |  | Fail | 0 | - | - | - | - | - | - | - | - |
| *2* | **Cereal-based foods** | Pass | 0 | - | - | - | - | - | - | - | - |
|  |  | Fail | 7 | 57.6 | 1.1 | 0.1 | 9.2 | 6.1 | 1.4 | 7.7 | 0.6 |
| *3* | **Complete meals** | Pass | 1 | 198.0 | 5.6 | 0.0 | 0.0 | 449.2 | 6.8 | 74.0 | 2.7 |
|  |  | Fail | 11 | 255.5 | 17.0 | 0.7 | 0.8 | 517.4 | 10.4 | 25.9 | 1.8 |
| *4* | **Center of plates** | Pass | 21 | 199.4 | 8.1 | 1.5 | 1.9 | 254.7 | 20.1 | 193.9 | 0.7 |
|  |  | Fail | 89 | 259.9 | 16.2 | 4.9 | 2.5 | 990.7 | 18.8 | 86.5 | 0.4 |
| *5* | **Small meals** | Pass | 5 | 225.0 | 6.6 | 1.1 | 2.0 | 185.2 | 6.6 | 7.8 | 0.9 |
|  |  | Fail | 11 | 260.0 | 9.7 | 2.5 | 5.6 | 276.8 | 9.8 | 26.5 | 1.3 |
| *6* | **Side dish** | Pass | 70 | 186.3 | 2.0 | 0.5 | 1.8 | 104.6 | 5.0 | 19.0 | 0.6 |
|  |  | Fail | 25 | 267.0 | 10.1 | 3.0 | 2.5 | 1610.9 | 6.5 | 51.6 | 1.9 |
| *7* | **Asian Noodles** | Pass | 3 | 186.6 | 3.9 | 0.4 | 0.0 | 115.5 | 4.5 | 27.1 | 0.7 |
|  |  | Fail | 6 | 312.7 | 14.2 | 7.4 | 19.5 | 576.7 | 9.3 | 27.7 | 0.4 |
| *8* | **Pizza as a center of plate** | Pass | 0 | - | - | - | - | - | - | - | - |
|  |  | Fail | 0 | - | - | - | - | - | - | - | - |
| *9* | **Soups** | Pass | 0 | - | - | - | - | - | - | - | - |
|  |  | Fail | 5 | 162.8 | 4.1 | 0.1 | 0.2 | 106.8 | 6.6 | 32.2 | 1.0 |
| *10* | **Cold cuts & spreads** | Pass | 4 | 125.8 | 5.4 | 1.8 | 1.7 | 244.0 | 17.8 | 4.3 | 0.0 |
|  |  | Fail | 21 | 367.0 | 21.3 | 6.8 | 2.9 | 1887.0 | 27.5 | 122.6 | 0.1 |
| *11* | **Salty & savoury snacks** | Pass | 11 | 415.6 | 13.6 | 4.2 | 4.6 | 203.4 | 8.6 | 92.3 | 1.8 |
|  |  | Fail | 8 | 528.4 | 31.9 | 9.3 | 10.1 | 314.0 | 7.6 | 22.5 | 2.9 |
| *12* | **Cheeses** | Pass | 1 | 426.0 | 15.0 | 11.9 | 0.0 | 79.3 | 55.1 | 730.0 | 0.0 |
|  |  | Fail | 6 | 297.7 | 18.4 | 17.0 | 0.0 | 608.3 | 24.4 | 656.0 | 0.0 |
| *13* | **Yoghurts & fresh cheeses** | Pass | 1 | 57.0 | 0.4 | 0.2 | 0.0 | 27.7 | 3.3 | 146.0 | 0.0 |
|  |  | Fail | 9 | 190.8 | 7.8 | 5.4 | 2.7 | 81.9 | 18.2 | 268.0 | 0.0 |
| *14* | **Dairy desserts** | Pass | 0 | - | - | - | - | - | - | - | - |
|  |  | Fail | 0 | - | - | - | - | - | - | - | - |
| *15* | **Ice creams** | Pass | 0 | - | - | - | - | - | - | - | - |
|  |  | Fail | 12 | 146.5 | 4.9 | 5.6 | 17.5 | 69.9 | 3.3 | 89.6 | 0.2 |
| *16* | **Low-fat ice creams** | Pass | 0 | - | - | - | - | - | - | - | - |
|  |  | Fail | 0 | - | - | - | - | - | - | - | - |
| *17* | **Water ice creams** | Pass | 0 | - | - | - | - | - | - | - | - |
|  |  | Fail | 0 | - | - | - | - | - | - | - | - |
| *18* | **Enriched Beverages** | Pass | 0 | - | - | - | - | - | - | - | - |
|  |  | Fail | 0 | - | - | - | - | - | - | - | - |
| *19* | **Culinary sauces** | Pass | 1 | 85.0 | 0.2 | 0.2 | 1.0 | 37.1 | 4.9 | 28.0 | 2.1 |
|  |  | Fail | 0 | - | - | - | - | - | - | - | - |
| *20* | **Milk-based beverages** | Pass | 10 | 179.7 | 8.4 | 3.4 | 0.5 | 75.5 | 8.0 | 298.2 | 0.0 |
|  |  | Fail | 39 | 71.4 | 2.6 | 1.4 | 2.9 | 59.2 | 3.2 | 130.3 | 0.1 |
| *21* | **Malt-based beverages** | Pass | 0 | - | - | - | - | - | - | - | - |
|  |  | Fail | 1 | 429.0 | 9.7 | 3.0 | 14.5 | 177.8 | 8.5 | 145.0 | 0.0 |
| *22* | **Cereal-based beverages** | Pass | 0 | - | - | - | - | - | - | - | - |
|  |  | Fail | 0 | - | - | - | - | - | - | - | - |
| *23* | **Confectionary bars** | Pass | 0 | - | - | - | - | - | - | - | - |
|  |  | Fail | 5 | 587.2 | 50.0 | 6.6 | 0.0 | 163.0 | 22.1 | 94.0 | 6.2 |
| *24* | **Chocolate** | Pass | 0 | - | - | - | - | - | - | - | - |
|  |  | Fail | 3 | 560.0 | 36.2 | 18.5 | 41.9 | 78.0 | 5.6 | 68.0 | 2.9 |
| *25* | **Juice-based beverages** | Pass | 7 | 43.6 | 0.1 | 0.0 | 0.0 | 5.2 | 0.3 | 6.0 | 0.1 |
|  |  | Fail | 10 | 105.4 | 0.1 | 0.0 | 8.2 | 56.5 | 0.5 | 12.9 | 0.1 |
| *26* | **Cakes, cookies & desserts** | Pass | 26 | 376.2 | 12.3 | 5.2 | 19.3 | 146.7 | 6.4 | 52.9 | 1.7 |
|  |  | Fail | 54 | 384.1 | 14.1 | 5.9 | 27.8 | 110.1 | 6.2 | 42.9 | 1.8 |
| *27* | **Beverages** | Pass | 19 | 190.7 | 1.4 | 0.1 | 0.0 | 15.7 | 13.3 | 222.5 | 9.9 |
|  |  | Fail | 20 | 146.2 | 0.4 | 0.4 | 5.8 | 14.8 | 0.3 | 15.7 | 0.1 |
| *28* | **Sugar confectionary** | Pass | 22 | 340.8 | 2.8 | 0.2 | 51.3 | 86.5 | 1.8 | 41.0 | 0.5 |
|  |  | Fail | 2 | 458.0 | 17.8 | 0.0 | 80.0 | 16.8 | 2.5 | 11.0 | 2.4 |
| *29* | **Dairy accessories** | Pass | 0 | - | - | - | - | - | - | - | - |
|  |  | Fail | 5 | 604.4 | 60.8 | 32.3 | 53.8 | 157.0 | 2.9 | 121.2 | 0.0 |
| *30* | **Dressings** | Pass | 10 | 86.4 | 4.5 | 0.3 | 1.3 | 294.9 | 3.8 | 125.6 | 0.0 |
|  |  | Fail | 28 | 851.9 | 92.5 | 28.5 | 0.0 | 303.0 | 1.9 | 32.4 | 0.0 |
| *31* | **Mayonnaise** | Pass | 0 | - | - | - | - | - | - | - | - |
|  |  | Fail | 0 | - | - | - | - | - | - | - | - |
| *32* | **Cold sauces** | Pass | 3 | 331.0 | 11.8 | 2.1 | 5.1 | 11.5 | 11.3 | 233.3 | 5.6 |
|  |  | Fail | 17 | 193.5 | 11.3 | 1.6 | 4.5 | 3168.6 | 9.2 | 209.8 | 3.1 |
| *33* | **Bouillons & seasonings** | Pass | 9 | 85.4 | 0.4 | 0.1 | 0.0 | 2739.0 | 6.3 | 51.3 | 0.1 |
|  |  | Fail | 3 | 60.0 | 0.3 | 0.1 | 0.0 | 5487.1 | 6.9 | 227.3 | 0.1 |
| *34* | **Culinary sauces as accessory** | Pass | 0 | - | - | - | - | - | - | - | - |
|  |  | Fail | 1 | 488.0 | 43.0 | 0.0 | 0.0 | 1260.5 | 6.2 | 35.0 | 0.0 |
| *35* | **Creamers** | Pass | 1 | 554.0 | 34.9 | 32.5 | 54.9 | 200.0 | 2.7 | 4.0 | 0.0 |
|  |  | Fail | 0 | - | - | - | - | - | - | - | - |

*Energy in kcal, all nutrients in gram/100g except for sodium and calcium in mg/100g.*

**TABLE S4a. Minimum reformulation (%) required to reach NNPS threshold in relevant products for each category – UK**

|  | **NNPS Categories** | **Energy (Kcal)** | **Total Fat** | **Saturated Fat** | **Added sugar** | **Sodium** | **Protein** | **Calcium** | **Fibre** |
| --- | --- | --- | --- | --- | --- | --- | --- | --- | --- |
| *1* | **Milk-based breakfast beverages** | - | -40% | -48% | - | - | - | - |  |
| *2* | **Cereal-based foods** | - | - | - | - | - | - | - | - |
| *3* | **Complete meals** | -11% | -27% | -26% | -27% | -21% | 1% |  |  |
| *4* | **Center of plates** | -12% | -27% | -21% | -21% | -29% | 4% |  |  |
| *5* | **Small meals** | -16% | -32% | -37% | -6% | -27% | 4% |  |  |
| *6* | **Side dish** | -8% | -28% | -20% | -26% | -18% |  |  |  |
| *7* | **Asian Noodles as main dish** | - | -16% | - | - | -15% |  |  |  |
| *8* | **Pizza as a center of plate** | -16% | -23% | -25% | - | -30% | 1% |  |  |
| *9* | **Soups** | -20% | -37% | -55% | -50% | -29% |  |  |  |
| *10* | **Cold cuts & spreads** | -30% | -47% | -51% | - | -60% |  |  |  |
| *11* | **Salty & savoury snacks** | - | -11% | -25% | - | -24% |  |  |  |
| *12* | **Cheeses** | - | -27% | -31% | - | -12% | - |  |  |
| *13* | **Yoghurts & fresh cheeses** | -10% | -30% | -33% | -16% | -20% | 1% | 199% |  |
| *14* | **Dairy desserts** | -11% | -20% | -35% | -39% | -27% | - | 30% |  |
| *15* | **Ice creams** | -27% | -42% | -51% | -22% | -6% |  |  |  |
| *16* | **Low-fat ice creams** | - | - | -1% | -3% | - |  |  |  |
| *17* | **Water ice creams** | - | - | - | - | - |  |  |  |
| *18* | **Enriched beverages** | - | - | - | - | - | - |  |  |
| *19* | **Culinary sauces** | -29% | -55% | -55% | -59% | -24% |  |  |  |
| *20* | **Milk-based beverages** | -2% | -7% | -10% | -5% | -4% | - | 66% |  |
| *21* | **Malt-based beverages** | -12% | -30% | -42% | - | - |  |  |  |
| *22* | **Cereal-based beverages** | - | - | - | - | - |  |  |  |
| *23* | **Confectionary bars** | -11% | -40% | -21% | -9% | -25% |  |  |  |
| *24* | **Chocolate** | -5% | -11% | -29% | -37% | - |  |  |  |
| *25* | **Juice-based beverages** | - | - | - | -91% | -40% |  |  |  |
| *26* | **Cakes, cookies & desserts** | -24% | -30% | -39% | -33% | -28% |  |  |  |
| *27* | **Beverages** | -2% | 0% | 0% | -9% | -1% |  |  |  |
| *28* | **Sugar confectionary** | -25% | -45% | -60% | -38% | -17% |  |  |  |
| *29* | **Dairy accessories** | -15% | -33% | -41% | -7% | -7% |  |  |  |
| *30* | **Dressings** | -19% | -45% | -61% | -41% | -23% |  |  |  |
| *31* | **Mayonnaise** | -9% | -5% | -45% | - | - |  |  |  |
| *32* | **Cold sauces** | - | - | -34% | -62% | -52% |  |  |  |
| *33* | **Bouillons & seasonings** | -32% | -58% | -41% | - | -20% |  |  |  |
| *34* | **Culinary sauces as accessory** | -58% | -41% | -78% | -60% | -55% |  |  |  |
| *35* | **Creamers** | -38% | -67% | -79% | - | - |  |  |  |

*Reformulation requirement is calculated for relevant products which did not meet the threshold for a given category and a given nutrient*

**TABLE S4b. Minimum reformulation (%) required to reach NNPS threshold in relevant products for each category – France**

| **CAT** | **NNPS Categories** | **Energy (Kcal)** | **Total Fat** | **Saturated Fat** | **Added sugar** | **Sodium** | **Protein** | **Calcium** | **Fibre** |
| --- | --- | --- | --- | --- | --- | --- | --- | --- | --- |
| *1* | **Milk-based breakfast beverages** | - | - | - | - | - | - | - |  |
| *2* | **Cereal-based foods** | 30% | - | - | - | - | 71% | 1609% | - |
| *3* | **Complete meals** | - | -27% | -27% | - | - | - |  |  |
| *4* | **Center of plates** | - | -24% | -23% | - | -27% | 34% |  |  |
| *5* | **Small meals** | -14% | -27% | -31% | -57% | -27% | -21% |  |  |
| *6* | **Side dish** | - | -23% | -6% | - | -23% |  |  |  |
| *7* | **Asian Noodles as main dish** | - | - | - | - | - |  |  |  |
| *8* | **Pizza as a center of plate** | - | -11% | - | - | -15% | - |  |  |
| *9* | **Soups** | - | -6% | -31% | -66% | -21% |  |  |  |
| *10* | **Cold cuts & spreads** | -21% | -42% | -50% | - | -49% |  |  |  |
| *11* | **Salty & savoury snacks** | - | -3% | -23% | - | -31% |  |  |  |
| *12* | **Cheeses** | - | -19% | -25% | - | -11% | 44% |  |  |
| *13* | **Yoghurts & fresh cheeses** | -18% | -31% | -36% | -42% | - | 0% | 24% |  |
| *14* | **Dairy desserts** | -33% | -55% | -61% | -34% | - | - | 7% |  |
| *15* | **Ice creams** | -19% | -20% | -40% | -31% | - |  |  |  |
| *16* | **Low-fat ice creams** | - | - | - | - | - |  |  |  |
| *17* | **Water ice creams** | -4% | - | - | -43% | - |  |  |  |
| *18* | **Enriched beverages** | - | - | - | - | - | - |  |  |
| *19* | **Culinary sauces** | -23% | -36% | -37% | -89% | -37% |  |  |  |
| *20* | **Milk-based beverages** | -27% | -17% | -30% | -44% | - | - | 60% |  |
| *21* | **Malt-based beverages** | - | - | - | - | - |  |  |  |
| *22* | **Cereal-based beverages** | - | - | - | - | - |  |  |  |
| *23* | **Confectionary bars** | - | -31% | -30% | -10% | -22% |  |  |  |
| *24* | **Chocolate** | -8% | -23% | -10% | -29% | - |  |  |  |
| *25* | **Juice-based beverages** | - | - | - | -97% | -46% |  |  |  |
| *26* | **Cakes, cookies & desserts** | -34% | -32% | -44% | -35% | -41% |  |  |  |
| *27* | **Beverages** | -8% | - | - | -39% | -38% |  |  |  |
| *28* | **Sugar confectionary** | -20% | -35% | - | -34% | - |  |  |  |
| *29* | **Dairy accessories** | -20% | -28% | -31% | -24% | - |  |  |  |
| *30* | **Dressings** | -17% | -40% | -55% | - | -7% |  |  |  |
| *31* | **Mayonnaise** | -7% | - | -60% | - | - |  |  |  |
| *32* | **Cold sauces** | -40% | -33% | -52% | -30% | -58% |  |  |  |
| *33* | **Bouillons & seasonings** | - | - | - | - | -25% |  |  |  |
| *34* | **Culinary sauces as accessory** | -25% | -49% | -64% | - | -20% |  |  |  |
| *35* | **Creamers** | - | - | - | - | - |  |  |  |

*Reformulation requirement is calculated for relevant products which did not meet the threshold for a given category and a given nutrient*

**TABLE S4c. Minimum reformulation (%) required to reach NNPS threshold in relevant products for each category – US**

| **CAT** | **NNPS Categories** | **Energy (Kcal)** | **Total Fat** | **Saturated Fat** | **Added sugar** | **Sodium** | **Protein** | **Calcium** | **Fibre** |
| --- | --- | --- | --- | --- | --- | --- | --- | --- | --- |
| 1 | **Milk-based breakfast beverages** | - | - | - | -1% | - | - | - |  |
| 2 | **Cereal-based foods** | 45% | -21% | -24% | -29% | -42% | 2% | 780% | 80% |
| 3 | **Complete meals** | -14% | -21% | -18% | - | -14% | 1% |  |  |
| 4 | **Center of plates** | -11% | -25% | -19% | -23% | -21% | 7% |  |  |
| 5 | **Small meals** | -11% | -21% | -15% | -30% | -23% | 1% |  |  |
| 6 | **Side dish** | -22% | -25% | -16% | -24% | -23% |  |  |  |
| 7 | **Asian Noodles as main dish** | -41% | -24% | - | - | -15% |  |  |  |
| 8 | **Pizza as a center of plate** | -12% | -12% | -13% | - | -12% | 0% |  |  |
| 9 | **Soups** | -25% | -34% | -36% | -40% | -14% |  |  |  |
| 10 | **Cold cuts & spreads** | -19% | -46% | -51% | -29% | -59% |  |  |  |
| 11 | **Salty & savoury snacks** | -12% | -8% | -36% | -22% | -17% |  |  |  |
| 12 | **Cheeses** | - | -21% | -26% | - | -15% | 1% |  |  |
| 13 | **Yoghurts & fresh cheeses** | -19% | -28% | -38% | -47% | -36% | 0% | 54% |  |
| 14 | **Dairy desserts** | -39% | -46% | -45% | -33% | -16% | 1% | 106% |  |
| 15 | **Ice creams** | -19% | -28% | -40% | -25% | -36% |  |  |  |
| 16 | **Low-fat ice creams** | -27% | -36% | -48% | -21% | -21% |  |  |  |
| 17 | **Water ice creams** | -6% | - | -48% | -37% | - |  |  |  |
| 18 | **Enriched beverages** | - | - | - | - | - | - |  |  |
| 19 | **Culinary sauces** | -28% | -39% | -40% | - | -15% |  |  |  |
| 20 | **Milk-based beverages** | -26% | -33% | -36% | -34% | -20% | 2% | 83% |  |
| 21 | **Malt-based beverages** | - | -16% | -9% | -7% | - |  |  |  |
| 22 | **Cereal-based beverages** | -3% | - | - | -52% | - |  |  | 150% |
| 23 | **Confectionary bars** | -4% | -37% | -29% | -17% | -21% |  |  |  |
| 24 | **Chocolate** | -5% | -17% | -30% | -29% | -18% |  |  |  |
| 25 | **Juice-based beverages** | -18% | -14% | -58% | -96% | -43% |  |  |  |
| 26 | **Cakes, cookies & desserts** | -30% | -31% | -38% | -35% | -37% |  |  |  |
| 27 | **Beverages** | -18% | -61% | -51% | -41% | -37% |  |  |  |
| 28 | **Sugar confectionary** | -12% | -47% | -39% | -34% | - |  |  |  |
| 29 | **Dairy accessories** | -18% | -34% | -27% | -33% | - |  |  |  |
| 30 | **Dressings** | -15% | -37% | -53% | -38% | -16% |  |  |  |
| 31 | **Mayonnaise** | -2% | - | -43% | -19% | -3% |  |  |  |
| 32 | **Cold sauces** | -12% | -19% | -54% | -45% | -40% |  |  |  |
| 33 | **Bouillons & seasonings** | -27% | -51% | -42% | -60% | -20% |  |  |  |
| 34 | **Culinary sauces as accessory** | -39% | -54% | -56% | -66% | -46% |  |  |  |
| 35 | **Creamers** | - | - | - | - | - |  |  |  |

*Reformulation requirement is calculated for relevant products which did not meet the threshold for a given category and a given nutrient*

**TABLE S4d. Minimum reformulation (%) required to reach NNPS threshold in relevant products for each category – Brazil**

| **CAT** | **NNPS Categories** | **Energy (Kcal)** | **Total Fat** | **Saturated Fat** | **Added sugar** | **Sodium** | **Protein** | **Calcium** | **Fibre** |
| --- | --- | --- | --- | --- | --- | --- | --- | --- | --- |
| **1** | **Milk-based breakfast beverages** | - | - | - | - | - | - | - | - |
| **2** | **Cereal-based foods** | 36% | -26% | - | - | -22% | 156% | 4212% | 59% |
| **3** | **Complete meals** | - | -24% | -25% | - | -35% | 79% |  |  |
| **4** | **Center of plates** | -23% | -36% | -29% | - | -44% | 175% |  |  |
| **5** | **Small meals** | -13% | -25% | -31% | -55% | -35% | 62% |  |  |
| **6** | **Side dish** | -16% | -25% | -28% | -53% | -10% |  |  |  |
| **7** | **Asian Noodles as main dish** | -42% | -33% | - | - | - |  |  |  |
| **8** | **Pizza as a center of plate** | - | -5% | -3% | - | -22% | - |  |  |
| **9** | **Soups** | -50% | -51% | -40% | -60% | -10% |  |  |  |
| **10** | **Cold cuts & spreads** | -16% | -45% | -55% | -89% | -59% |  |  |  |
| **11** | **Salty & savoury snacks** | - | -1% | - | -42% | -7% |  |  |  |
| **12** | **Cheeses** | - | -12% | -19% | - | -21% | - |  |  |
| **13** | **Yoghurts & fresh cheeses** | -17% | -44% | -49% | -45% | -47% | 39% | 132% |  |
| **14** | **Dairy desserts** | -44% | -48% | -52% | -45% | -40% | 33% | 242% |  |
| **15** | **Ice creams** | - | -2% | -30% | -26% | - |  |  |  |
| **16** | **Low-fat ice creams** | - | - | - | -15% | - |  |  |  |
| **17** | **Water ice creams** | -3% | - | - | -32% | - |  |  |  |
| **18** | **Enriched beverages** | - | - | - | - | - | - |  |  |
| **19** | **Culinary sauces** | -41% | -61% | -60% | - | -25% |  |  |  |
| **20** | **Milk-based beverages** | -28% | -23% | -26% | -31% | -51% | 23% | 88% |  |
| **21** | **Malt-based beverages** | - | - | - | - | - |  |  |  |
| **22** | **Cereal-based beverages** | - | - | - | - | - |  |  |  |
| **23** | **Confectionary bars** | - | -27% | -2% | -31% | - |  |  |  |
| **24** | **Chocolate** | -7% | -20% | -47% | -32% | - |  |  |  |
| **25** | **Juice-based beverages** | -12% | -50% | -54% | -97% | -90% |  |  |  |
| **26** | **Cakes, cookies & desserts** | -21% | -20% | -36% | -35% | -25% |  |  |  |
| **27** | **Beverages** | -74% | -48% | -17% | - | -61% |  |  |  |
| **28** | **Sugar confectionary** | -29% | -31% | -63% | -29% | - |  |  |  |
| **29** | **Dairy accessories** | - | -62% | -66% | - | - |  |  |  |
| **30** | **Dressings** | 0% | -38% | -61% | - | -38% |  |  |  |
| **31** | **Mayonnaise** | - | - | - | -27% | -6% |  |  |  |
| **32** | **Cold sauces** | - | - | - | -54% | -67% |  |  |  |
| **33** | **Bouillons & seasonings** | - | - | - | - | -15% |  |  |  |
| **34** | **Culinary sauces as accessory** | - | -44% | -73% | - | - |  |  |  |
| **35** | **Creamers** | - | - | - | - | - |  |  |  |

*Reformulation requirement is calculated for relevant products which did not meet the threshold for a given category and a given nutrient*

**TABLE S4e. Minimum reformulation (%) required to reach NNPS threshold in relevant products for each category – China**

| **CAT** | **NNPS Categories** | **Energy (Kcal)** | **Total Fat** | **Saturated Fat** | **Added sugar** | **Sodium** | **Protein** | **Calcium** | **Fibre** |
| --- | --- | --- | --- | --- | --- | --- | --- | --- | --- |
| **1** | **Milk-based breakfast beverages** | - | - | - | - | - | - | - |  |
| **2** | **Cereal-based foods** | 55% | -39% | - | -81% | - | 274% | 1707% | 632% |
| **3** | **Complete meals** | -15% | -37% | -45% | - | -26% | 8% |  |  |
| **4** | **Center of plates** | -21% | -29% | -23% | -30% | -42% | 932% |  |  |
| **5** | **Small meals** | -4% | -18% | -5% | -66% | -7% | 67% |  |  |
| **6** | **Side dish** | -21% | -33% | -54% | -75% | -76% |  |  |  |
| **7** | **Asian Noodles as main dish** | -34% | -13% | -38% | - | -53% |  |  |  |
| **8** | **Pizza as a center of plate** | - | - | - | - | - | - |  |  |
| **9** | **Soups** | -41% | -53% | - | - | - |  |  |  |
| **10** | **Cold cuts & spreads** | -25% | -50% | -57% | -25% | -56% |  |  |  |
| **11** | **Salty & savoury snacks** | - | -15% | -33% | -77% | - |  |  |  |
| **12** | **Cheeses** | - | -16% | -24% | - | -17% | - |  |  |
| **13** | **Yoghurts & fresh cheeses** | -18% | -38% | -44% | -59% | - | 9% | 114% |  |
| **14** | **Dairy desserts** | - | - | - | - | - | - | - |  |
| **15** | **Ice creams** | - | -9% | -30% | -15% | -24% |  |  |  |
| **16** | **Low-fat ice creams** | - | - | - | - | - |  |  |  |
| **17** | **Water ice creams** | - | - | - | - | - |  |  |  |
| **18** | **Enriched beverages** | - | - | - | - | - | - |  |  |
| **19** | **Culinary sauces** | - | - | - | - | - |  |  |  |
| **20** | **Milk-based beverages** | -82% | -25% | -28% | -34% | -70% | 761% | 1836% |  |
| **21** | **Malt-based beverages** | -81% | -77% | -58% | -64% | -44% |  |  |  |
| **22** | **Cereal-based beverages** | - | - | - | - | - |  |  |  |
| **23** | **Confectionary bars** | - | -53% | -21% | - | - |  |  |  |
| **24** | **Chocolate** | -10% | -26% | -46% | -25% | - |  |  |  |
| **25** | **Juice-based beverages** | -38% | - | - | -98% | -59% |  |  |  |
| **26** | **Cakes, cookies & desserts** | -31% | -27% | -35% | -49% | -34% |  |  |  |
| **27** | **Beverages** | -40% | -9% | -65% | -42% | -17% |  |  |  |
| **28** | **Sugar confectionary** | - | -34% | - | -14% | - |  |  |  |
| **29** | **Dairy accessories** | -41% | -83% | -82% | -26% | - |  |  |  |
| **30** | **Dressings** | -19% | -49% | -64% | - | -82% |  |  |  |
| **31** | **Mayonnaise** | - | - | - | - | - |  |  |  |
| **32** | **Cold sauces** | -42% | -54% | -55% | -78% | -85% |  |  |  |
| **33** | **Bouillons & seasonings** | - | - | - | - | -12% |  |  |  |
| **34** | **Culinary sauces as accessory** | -69% | -88% | - | - | -64% |  |  |  |
| **35** | **Creamers** | - | - | - | - | - |  |  |  |

*Reformulation requirement is calculated for relevant products which did not meet the threshold for a given category and a given nutrient*

**TABLE S5a. Changes to the nutrient composition of all products in scope (%) when minimum reformulation required to reach NNPS threshold is applied- UK**

|  | **NNPS Categories** | **Energy (Kcal)** | **Total Fat** | **Saturated Fat** | **Added sugar** | **Sodium** | **Protein** | **Calcium** | **Fibre** |
| --- | --- | --- | --- | --- | --- | --- | --- | --- | --- |
| **1** | **Milk-based breakfast beverages** | 0% | -40% | -48% | 0% | 0% | 0% | 0% |  |
| **2** | **Cereal-based foods** | - | - | - | - | - | - | - | - |
| **3** | **Complete meals** | -1% | -18% | -12% | -1% | -2% | 0% |  |  |
| **4** | **Center of plates** | -1% | -15% | -6% | 0% | -4% | 1% |  |  |
| **5** | **Small meals** | -4% | -26% | -14% | 0% | -7% | 2% |  |  |
| **6** | **Side dish** | 0% | -7% | -2% | -1% | -2% |  |  |  |
| **7** | **Asian Noodles as main dish** | 0% | -11% | 0% | 0% | -10% |  |  |  |
| **8** | **Pizza as a center of plate** | -13% | -9% | -5% | 0% | -6% | 0% |  |  |
| **9** | **Soups** | -4% | -27% | -23% | -38% | -19% |  |  |  |
| **10** | **Cold cuts & spreads** | -3% | -28% | -33% | 0% | -53% |  |  |  |
| **11** | **Salty & savoury snacks** | 0% | -2% | -8% | 0% | -11% |  |  |  |
| **12** | **Cheeses** | 0% | -21% | -27% | 0% | -2% | 0% |  |  |
| **13** | **Yoghurts & fresh cheeses** | -2% | -13% | -16% | -3% | -8% | 0% | 72% |  |
| **14** | **Dairy desserts** | -2% | -7% | -6% | -11% | -4% | 0% | 5% |  |
| **15** | **Ice creams** | -12% | -21% | -38% | -14% | -1% |  |  |  |
| **16** | **Low-fat ice creams** | 0% | 0% | 0% | -2% | 0% |  |  |  |
| **17** | **Water ice creams** | - | - | - | - | - |  |  |  |
| **18** | **Enriched beverages** | - | - | - | - | - | - |  |  |
| **19** | **Culinary sauces** | -20% | -46% | -37% | -37% | -11% |  |  |  |
| **20** | **Milk-based beverages** | -1% | -3% | -4% | -2% | -1% | 0% | 5% |  |
| **21** | **Malt-based beverages** | -6% | -15% | -21% | 0% | 0% |  |  |  |
| **22** | **Cereal-based beverages** | - | - | - | - | - |  |  |  |
| **23** | **Confectionary bars** | -1% | -36% | -14% | -1% | -3% |  |  |  |
| **24** | **Chocolate** | -2% | -5% | -24% | -37% | 0% |  |  |  |
| **25** | **Juice-based beverages** | 0% | 0% | 0% | -27% | -3% |  |  |  |
| **26** | **Cakes, cookies & desserts** | -12% | -10% | -20% | -18% | -8% |  |  |  |
| **27** | **Beverages** | -1% | 0% | 0% | -7% | 0% |  |  |  |
| **28** | **Sugar confectionary** | -12% | -10% | -13% | -27% | -1% |  |  |  |
| **29** | **Dairy accessories** | -4% | -20% | -25% | -1% | -1% |  |  |  |
| **30** | **Dressings** | -12% | -33% | -47% | -5% | -2% |  |  |  |
| **31** | **Mayonnaise** | -6% | -2% | -30% | 0% | 0% |  |  |  |
| **32** | **Cold sauces** | 0% | 0% | -4% | -29% | -20% |  |  |  |
| **33** | **Bouillons & seasonings** | -2% | -4% | -3% | 0% | -6% |  |  |  |
| **34** | **Culinary sauces as accessory** | -7% | -15% | -20% | -38% | -20% |  |  |  |
| **35** | **Creamers** | -38% | -67% | -79% | 0% | 0% |  |  |  |

*Changes to the nutrient composition of all products in scope is calculated for all products in each category and for each nutrient, regardless of whether threshold was originally met.*

**TABLE S5b. Changes to the nutrient composition of all products in scope (%) when minimum reformulation required to reach NNPS threshold is applied- France**

| **CAT** | **NNPS Categories** | **Energy (Kcal)** | **Total Fat** | **Saturated Fat** | **Added sugar** | **Sodium** | **Protein** | **Calcium** | **Fibre** |
| --- | --- | --- | --- | --- | --- | --- | --- | --- | --- |
| **1** | **Milk-based breakfast beverages** | - | - | - | - | - | - | - |  |
| **2** | **Cereal-based foods** | 30% | 0% | 0% | 0% | 0% | 71% | 1609% | 0% |
| **3** | **Complete meals** | 0% | -13% | -10% | 0% | 0% | 0% |  |  |
| **4** | **Center of plates** | 0% | -12% | -9% | 0% | -6% | 6% |  |  |
| **5** | **Small meals** | -5% | -17% | -11% | -1% | -15% | -2% |  |  |
| **6** | **Side dish** | 0% | -3% | 0% | 0% | -3% |  |  |  |
| **7** | **Asian Noodles as main dish** | - | - | - | - | - |  |  |  |
| **8** | **Pizza as a center of plate** | 0% | -1% | 0% | 0% | -2% | 0% |  |  |
| **9** | **Soups** | 0% | 0% | -8% | -4% | -7% |  |  |  |
| **10** | **Cold cuts & spreads** | -2% | -29% | -29% | 0% | -43% |  |  |  |
| **11** | **Salty & savoury snacks** | 0% | 0% | -9% | 0% | -2% |  |  |  |
| **12** | **Cheeses** | 0% | -13% | -20% | 0% | -1% | 3% |  |  |
| **13** | **Yoghurts & fresh cheeses** | -6% | -9% | -11% | -14% | 0% | 0% | 13% |  |
| **14** | **Dairy desserts** | -7% | -11% | -13% | -29% | 0% | 0% | 1% |  |
| **15** | **Ice creams** | -10% | -10% | -40% | -31% | 0% |  |  |  |
| **16** | **Low-fat ice creams** | - | - | - | - | - |  |  |  |
| **17** | **Water ice creams** | -4% | 0% | 0% | -43% | 0% |  |  |  |
| **18** | **Enriched beverages** | - | - | - | - | - | - |  |  |
| **19** | **Culinary sauces** | -2% | -23% | -20% | -8% | -34% |  |  |  |
| **20** | **Milk-based beverages** | -2% | -1% | -2% | -17% | 0% | 0% | 23% |  |
| **21** | **Malt-based beverages** | 0% | 0% | 0% | 0% | 0% |  |  |  |
| **22** | **Cereal-based beverages** | - | - | - | - | - |  |  |  |
| **23** | **Confectionary bars** | 0% | -10% | -11% | -3% | -6% |  |  |  |
| **24** | **Chocolate** | -5% | -13% | -9% | -25% | 0% |  |  |  |
| **25** | **Juice-based beverages** | 0% | 0% | 0% | -28% | -7% |  |  |  |
| **26** | **Cakes, cookies & desserts** | -21% | -15% | -31% | -18% | -17% |  |  |  |
| **27** | **Beverages** | -1% | 0% | 0% | -7% | -4% |  |  |  |
| **28** | **Sugar confectionary** | -2% | -2% | 0% | -8% | 0% |  |  |  |
| **29** | **Dairy accessories** | -1% | -5% | -8% | -2% | 0% |  |  |  |
| **30** | **Dressings** | -11% | -33% | -51% | 0% | 0% |  |  |  |
| **31** | **Mayonnaise** | -5% | 0% | -45% | 0% | 0% |  |  |  |
| **32** | **Cold sauces** | -9% | -15% | -29% | -7% | -45% |  |  |  |
| **33** | **Bouillons & seasonings** | 0% | 0% | 0% | 0% | -15% |  |  |  |
| **34** | **Culinary sauces as accessory** | -6% | -30% | -32% | 0% | -5% |  |  |  |
| **35** | **Creamers** | - | - | - | - | - |  |  |  |

*Changes to the nutrient composition of all products in scope is calculated for all products in each category and for each nutrient, regardless of whether threshold was originally met.*

**TABLE S5c. Changes to the nutrient composition of all products in scope (%) when minimum reformulation required to reach NNPS threshold is applied- US**

| **CAT** | **NNPS Categories** | **Energy (Kcal)** | **Total Fat** | **Saturated Fat** | **Added sugar** | **Sodium** | **Protein** | **Calcium** | **Fibre** |
| --- | --- | --- | --- | --- | --- | --- | --- | --- | --- |
| **1** | **Milk-based breakfast beverages** | 0% | 0% | 0% | -1% | 0% | 0% | 0% |  |
| **2** | **Cereal-based foods** | 23% | -7% | -3% | -1% | -36% | 1% | 685% | 36% |
| **3** | **Complete meals** | 0% | -8% | -3% | 0% | -1% | 0% |  |  |
| **4** | **Center of plates** | 0% | -10% | -3% | 0% | -4% | 1% |  |  |
| **5** | **Small meals** | -3% | -14% | -3% | -1% | -13% | 0% |  |  |
| **6** | **Side dish** | 0% | -4% | -1% | -1% | -3% |  |  |  |
| **7** | **Asian Noodles as main dish** | -2% | -9% | 0% | 0% | -2% |  |  |  |
| **8** | **Pizza as a center of plate** | -2% | -5% | -4% | 0% | -7% | 0% |  |  |
| **9** | **Soups** | -3% | -10% | -15% | -3% | -6% |  |  |  |
| **10** | **Cold cuts & spreads** | -3% | -24% | -24% | -2% | -49% |  |  |  |
| **11** | **Salty & savoury snacks** | 0% | -1% | -1% | -2% | -1% |  |  |  |
| **12** | **Cheeses** | 0% | -11% | -14% | 0% | -2% | 0% |  |  |
| **13** | **Yoghurts & fresh cheeses** | -8% | -5% | -6% | -15% | -4% | 0% | 25% |  |
| **14** | **Dairy desserts** | -6% | -8% | -8% | -19% | -3% | 0% | 46% |  |
| **15** | **Ice creams** | -8% | -12% | -25% | -16% | -2% |  |  |  |
| **16** | **Low-fat ice creams** | -4% | -4% | -8% | -11% | -3% |  |  |  |
| **17** | **Water ice creams** | -3% | 0% | -8% | -19% | 0% |  |  |  |
| **18** | **Enriched beverages** | - | - | - | - | - |  |  |  |
| **19** | **Culinary sauces** | -5% | -18% | -11% | 0% | -10% |  |  |  |
| **20** | **Milk-based beverages** | -5% | -5% | -6% | -12% | -1% | 0% | 14% |  |
| **21** | **Malt-based beverages** | 0% | -8% | -9% | -4% | 0% |  |  |  |
| **22** | **Cereal-based beverages** | -2% | 0% | 0% | -52% | 0% |  |  | 113% |
| **23** | **Confectionary bars** | 0% | -18% | -12% | -4% | -7% |  |  |  |
| **24** | **Chocolate** | -2% | -6% | -18% | -21% | -2% |  |  |  |
| **25** | **Juice-based beverages** | -1% | 0% | -2% | -35% | -7% |  |  |  |
| **26** | **Cakes, cookies & desserts** | -15% | -13% | -14% | -15% | -19% |  |  |  |
| **27** | **Beverages** | -3% | -1% | -1% | -14% | -3% |  |  |  |
| **28** | **Sugar confectionary** | -3% | -3% | -6% | -11% | 0% |  |  |  |
| **29** | **Dairy accessories** | -1% | -5% | -8% | -1% | 0% |  |  |  |
| **30** | **Dressings** | -7% | -23% | -35% | -9% | -5% |  |  |  |
| **31** | **Mayonnaise** | 0% | 0% | -5% | -2% | -1% |  |  |  |
| **32** | **Cold sauces** | 0% | -3% | -12% | -10% | -22% |  |  |  |
| **33** | **Bouillons & seasonings** | -5% | -10% | -5% | -7% | -6% |  |  |  |
| **34** | **Culinary sauces as accessory** | -11% | -26% | -25% | -21% | -25% |  |  |  |
| **35** | **Creamers** | 0% | 0% | 0% | 0% | 0% |  |  |  |

*Changes to the nutrient composition of all products in scope is calculated for all products in each category and for each nutrient, regardless of whether threshold was originally met.*

**TABLE S5d. Changes to the nutrient composition of all products in scope (%) when minimum reformulation required to reach NNPS threshold is applied - Brazil**

| **CAT** | **NNPS Categories** | **Energy (Kcal)** | **Total Fat** | **Saturated Fat** | **Added sugar** | **Sodium** | **Protein** | **Calcium** | **Fibre** |
| --- | --- | --- | --- | --- | --- | --- | --- | --- | --- |
| **1** | **Milk-based breakfast beverages** | - | - | - | - | - | - | - |  |
| **2** | **Cereal-based foods** | 9% | -13% | 0% | 0% | -11% | 156% | 4212% | 15% |
| **3** | **Complete meals** | 0% | -13% | -10% | 0% | -8% | 4% |  |  |
| **4** | **Center of plates** | -5% | -20% | -8% | 0% | -5% | 15% |  |  |
| **5** | **Small meals** | -5% | -15% | -9% | -1% | -16% | 6% |  |  |
| **6** | **Side dish** | 0% | -3% | -2% | -2% | 0% |  |  |  |
| **7** | **Asian Noodles as main dish** | -8% | -7% | 0% | 0% | 0% |  |  |  |
| **8** | **Pizza as a center of plate** | 0% | -1% | -2% | 0% | -19% | 0% |  |  |
| **9** | **Soups** | -9% | -11% | -13% | -18% | -5% |  |  |  |
| **10** | **Cold cuts & spreads** | -3% | -27% | -35% | -4% | -50% |  |  |  |
| **11** | **Salty & savoury snacks** | 0% | 0% | 0% | -23% | -1% |  |  |  |
| **12** | **Cheeses** | 0% | -5% | -10% | 0% | -5% | 0% |  |  |
| **13** | **Yoghurts & fresh cheeses** | -4% | -31% | -34% | -9% | -7% | 2% | 66% |  |
| **14** | **Dairy desserts** | -9% | -10% | -10% | -27% | -8% | 7% | 48% |  |
| **15** | **Ice creams** | 0% | -1% | -30% | -13% | 0% |  |  |  |
| **16** | **Low-fat ice creams** | 0% | 0% | 0% | -15% | 0% |  |  |  |
| **17** | **Water ice creams** | -1% | 0% | 0% | -32% | 0% |  |  |  |
| **18** | **Enriched beverages** | - | - | - | - | - | - |  |  |
| **19** | **Culinary sauces** | -10% | -15% | -15% | 0% | -13% |  |  |  |
| **20** | **Milk-based beverages** | -9% | -11% | -12% | -14% | -2% | 3% | 40% |  |
| **21** | **Malt-based beverages** | - | - | - | - | - |  |  |  |
| **22** | **Cereal-based beverages** | - | - | - | - | - |  |  |  |
| **23** | **Confectionary bars** | 0% | -7% | 0% | -19% | 0% |  |  |  |
| **24** | **Chocolate** | -5% | -18% | -47% | -21% | 0% |  |  |  |
| **25** | **Juice-based beverages** | 0% | -2% | -2% | -23% | -6% |  |  |  |
| **26** | **Cakes, cookies & desserts** | -11% | -4% | -13% | -19% | -5% |  |  |  |
| **27** | **Beverages** | -13% | -3% | -2% | 0% | -14% |  |  |  |
| **28** | **Sugar confectionary** | -5% | -8% | -9% | -11% | 0% |  |  |  |
| **29** | **Dairy accessories** | 0% | -21% | -22% | 0% | 0% |  |  |  |
| **30** | **Dressings** | 0% | -19% | -51% | 0% | -6% |  |  |  |
| **31** | **Mayonnaise** | 0% | 0% | 0% | -27% | -6% |  |  |  |
| **32** | **Cold sauces** | 0% | 0% | 0% | -36% | -45% |  |  |  |
| **33** | **Bouillons & seasonings** | 0% | 0% | 0% | 0% | -10% |  |  |  |
| **34** | **Culinary sauces as accessory** | 0% | -44% | -73% | 0% | 0% |  |  |  |
| **35** | **Creamers** | - | - | - | - | - |  |  |  |

*Changes to the nutrient composition of all products in scope is calculated for all products in each category and for each nutrient, regardless of whether threshold was originally met.*

**TABLE S5e. Changes to the nutrient composition of all products in scope (%) when minimum reformulation required to reach NNPS threshold is applied- China**

| **CAT** | **NNPS Categories** | **Energy (Kcal)** | **Total Fat** | **Saturated Fat** | **Added sugar** | **Sodium** | **Protein** | **Calcium** | **Fibre** |
| --- | --- | --- | --- | --- | --- | --- | --- | --- | --- |
| **1** | **Milk-based breakfast beverages** | - | - | - | - | - | - | - |  |
| **2** | **Cereal-based foods** | 55% | -6% | 0% | -35% | 0% | 274% | 1707% | 542% |
| **3** | **Complete meals** | -1% | -34% | -4% | 0% | -2% | 1% |  |  |
| **4** | **Center of plates** | -3% | -14% | -6% | 0% | -18% | 271% |  |  |
| **5** | **Small meals** | -1% | -6% | 0% | -4% | -1% | 21% |  |  |
| **6** | **Side dish** | -2% | -3% | -2% | -1% | -8% |  |  |  |
| **7** | **Asian Noodles as main dish** | -20% | -7% | -16% | 0% | -15% |  |  |  |
| **8** | **Pizza as a center of plate** | - | - | - | - | - | - |  |  |
| **9** | **Soups** | -33% | -32% | 0% | 0% | 0% |  |  |  |
| **10** | **Cold cuts & spreads** | -5% | -22% | -21% | -5% | -43% |  |  |  |
| **11** | **Salty & savoury snacks** | 0% | -3% | -7% | -8% | 0% |  |  |  |
| **12** | **Cheeses** | 0% | -5% | -17% | 0% | -2% | 0% |  |  |
| **13** | **Yoghurts & fresh cheeses** | -2% | -15% | -22% | -12% | 0% | 1% | 34% |  |
| **14** | **Dairy desserts** | - | - | - | - | - | - | - |  |
| **15** | **Ice creams** | 0% | -1% | -22% | -15% | -4% |  |  |  |
| **16** | **Low-fat ice creams** | - | - | - | - | - |  |  |  |
| **17** | **Water ice creams** | - | - | - | - | - |  |  |  |
| **18** | **Enriched beverages** | - | - | - | - | - | - |  |  |
| **19** | **Culinary sauces** | 0% | 0% | 0% | 0% | 0% |  |  |  |
| **20** | **Milk-based beverages** | -3% | -8% | -9% | -6% | -6% | 124% | 1049% |  |
| **21** | **Malt-based beverages** | -81% | -77% | -58% | -64% | -44% |  |  |  |
| **22** | **Cereal-based beverages** | - | - | - | - | - |  |  |  |
| **23** | **Confectionary bars** | 0% | -53% | -12% | 0% | 0% |  |  |  |
| **24** | **Chocolate** | -10% | -26% | -46% | -25% | 0% |  |  |  |
| **25** | **Juice-based beverages** | -13% | 0% | 0% | -23% | -7% |  |  |  |
| **26** | **Cakes, cookies & desserts** | -17% | -9% | -18% | -24% | -2% |  |  |  |
| **27** | **Beverages** | -16% | 0% | -5% | -13% | -1% |  |  |  |
| **28** | **Sugar confectionary** | 0% | -1% | 0% | -1% | 0% |  |  |  |
| **29** | **Dairy accessories** | -41% | -66% | -66% | -26% | 0% |  |  |  |
| **30** | **Dressings** | -13% | -33% | -45% | 0% | -2% |  |  |  |
| **31** | **Mayonnaise** | - | - | - | - | - |  |  |  |
| **32** | **Cold sauces** | -6% | -8% | -8% | -8% | -64% |  |  |  |
| **33** | **Bouillons & seasonings** | 0% | 0% | 0% | 0% | -3% |  |  |  |
| **34** | **Culinary sauces as accessory** | -69% | -88% | 0% | 0% | -64% |  |  |  |
| **35** | **Creamers** | 0% | 0% | 0% | 0% | 0% |  |  |  |

*Changes to the nutrient composition of all products in scope is calculated for all products in each category and for each nutrient, regardless of whether threshold was originally met.*
